# Interaction networks within disease-associated Gα_S_ variants characterized by an integrative biophysical approach

**DOI:** 10.1016/j.jbc.2024.107497

**Published:** 2024-06-24

**Authors:** Kara Anazia, Lucien Koenekoop, Guillaume Ferré, Enzo Petracco, Hugo Gutiérrez-de-Terán, Matthew T. Eddy

**Affiliations:** 1 Department of Chemistry, University of Florida, Gainesville, Florida, USA; 2 Department of Cell and Molecular Biology, Uppsala University, Uppsala, Sweden; 3 URD Agro-Biotechnologies Industrielles (ABI), CEBB, AgroParisTech, Pomacle, France

**Keywords:** GTPase, G protein, G protein–coupled receptor (GPCR), nuclear magnetic resonance (NMR), molecular dynamics (MD), cancer

## Abstract

Activation of G proteins through nucleotide exchange initiates intracellular signaling cascades essential for life processes. Under normal conditions, nucleotide exchange is regulated by the formation of G protein–G protein–coupled receptor complexes. Single point mutations in the Gα subunit of G proteins bypass this interaction, leading to loss of function or constitutive gain of function, which is closely linked with the onset of multiple diseases. Despite the recognized significance of Gα mutations in disease pathology, structural information for most variants is lacking, potentially due to inherent protein dynamics that pose challenges for crystallography. To address this, we leveraged an integrative spectroscopic and computational approach to structurally characterize seven of the most frequently observed and clinically relevant mutations in the stimulatory Gα subunit, Gα_S_. A previously proposed allosteric model of Gα activation linked structural changes in the nucleotide-binding pocket with functionally important changes in interactions between switch regions. We investigated this allosteric connection in Gα_S_ by integrating data from variable temperature CD spectroscopy, which measured changes in global protein structure and stability, and molecular dynamics simulations, which observed changes in interaction networks between Gα_S_ switch regions. Additionally, saturation-transfer difference NMR spectroscopy was applied to observe changes in nucleotide interactions with residues within the nucleotide binding site. These data have enabled testing of predictions regarding how mutations in Gα_S_ result in loss or gain of function and evaluation of proposed structural mechanisms. The integration of experimental and computational data allowed us to propose a more nuanced classification of mechanisms underlying Gα_S_ gain-of-function and loss-of-function mutations.

Signaling at the surface of most eukaryotic cells is triggered by the interaction of agonist-stimulated G protein–coupled receptors (GPCRs) with intracellular G proteins. GPCR–G protein complex formation accelerates the exchange of bound GDP for GTP, dissociating the trimeric G protein complex into α and βγ subunits. Both subunits then activate intracellular pathways until GTP is hydrolyzed, enabling the dissociated subunits to reassemble and restart the process (1, 2). In typical physiological conditions, nucleotide exchange and the cyclical dissociation and reassociation of G proteins is carefully controlled through interactions of G proteins with partner GPCRs and regulators of G protein signaling proteins. Acquired or inherited single point mutations, or residue-specific modifications, within the Gα subunit of the trimeric G protein subvert this process. Certain single point mutations within the Gα subunit accelerate GDP release or GTP uptake, causing constitutive activation and uncontrolled generation of secondary messengers (3, 4). Other mutations prevent GDP release or dissociation of the Gα and Gβγ subunits, irreversibly halting secondary messenger production (5). In both cases, circumventing regulation of G protein signaling is closely associated with the onset of a wide range of diseases (6, 7, 8), including inflammatory diseases (9), cardiovascular diseases (10, 11), metabolic disorders (12, 13), and cancers (14, 15, 16, 17).

The prevalence of disease-associated mutations in multiple Gα subtypes has prompted investigation of the structural mechanisms by which these mutations cause abnormal signaling. Literature data from integrative biophysical and biochemical experiments of the inhibitory Gα protein, Gα_i_, indicated the presence of a function-related allosteric network connecting the nucleotide-binding pocket and Gα_i_ “switch” regions (18, 19), structural features conserved among Gα proteins that undergo conformational changes upon nucleotide exchange (20). The presence of this allosteric network, and the correlation between changes in function with changes in hydrogen bonding between switch regions, also underlies a rational basis for explaining the impacts of disease-associated mutations in Gα_S_ (21). A crystal structure of the Gα_S_ R201C variant bound to GDP revealed a pattern of hydrogen bonding interactions among crucial switch regions similar to those in GTP-bound Gα_S_ (21). This indicated the structure of the GDP-bound R201C variant represented an active conformation, providing a plausible rationale for the gain of function observed for this variant (21). Additionally, the same study proposed that the loss of hydrogen bonding for the GTP-bound Gα_S_ variant R228C could explain the loss of function for this disease-associated variant (21). These findings raised the question of the extent to which changes in protein stability and changes in hydrogen bonding within the switch regions correlated with functional changes in other Gα_S_ variants. However, data on these aspects are lacking for most Gα_S_ variants. No structures are available for other Gα_S_ variants, and even for variants that have been structurally characterized, no structural information is available for both GDP and GTP complexes. It thus seemed pertinent to systematically measure these properties across a wider range of Gα_S_ variants.

Motivated by the lack of data measuring stability and hydrogen bonding for most Gα_S_ variants, we leveraged an integrative experimental and computational biophysical approach to characterize the structure and stability of seven of the most prevalent mutations in Gα_S_ and their influence on Gα_S_ structure-function relationships. Our studies were carried out in aqueous solutions, circumventing the need to crystallize the variants, which can be challenging. Using variable-temperature CD spectroscopy, we measured the global structural fold and stability for all variants. Changes in networks of hydrogen bonding and nonhydrogen bonding interactions were observed through molecular dynamics (MD) simulations, which enabled visualization of changes in interactions among the crucial switch regions. To observe the allosteric effects of mutations on the nucleotide-binding pocket, we employed saturation-transfer difference NMR (STD-NMR) spectroscopy in aqueous solutions to examine how mutations impacted the interaction of nucleotides with residues in the nucleotide-binding pocket and provide a structural and dynamic interpretation of this data. These data were compared with predictions made in earlier studies of Gα_S_, which enabled us to evaluate and revise previously proposed mechanisms of how disease-associated mutations alter Gα_S_ function. Given the occurrence of analogous mutations in other Gα subtypes, the mechanisms explored in this work may extend beyond Gα_S_.

## Results

### Prediction of properties of human Gα_S_ variants

**Figure 1:**
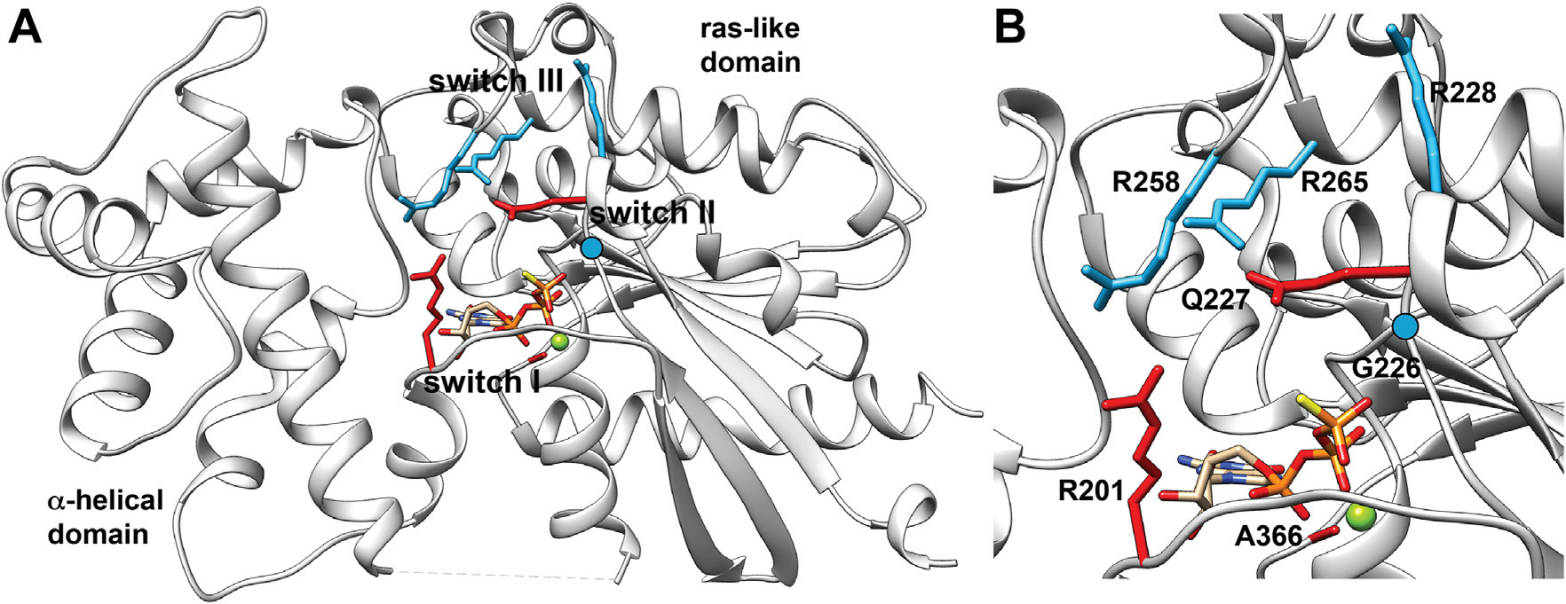
**Crystal structure of Gα**_**S**_**with annotated disease-associated mutations.***A*, the GTP-bound Gα_S_ crystal structure (PDB: 1AZT) (40). GTP is shown in *stick representation* and disease-associated mutations shown to increase or decrease adenylyl cyclase activity is shown in *cyan or red stick representation*, respectively. G226 is shown as a *cyan circle*. Magnesium is shown as a *green sphere*. *B*, expanded view of the nucleotide-binding pocket and annotated mutations of interest. Same presentation details as *A*.

Our study compared the biophysical and structural properties of Gα_S_ and 7 Gα_S_ variants containing single amino acid replacements: R201C, G226A, Q227L, R228C, R258A, R265H, and A366S (Fig. 1). These variants were selected because of their disease relevance (Table S1) and because alterations to their adenylate cyclase activity had been previously measured (Table S1). This allowed us to propose a framework for relating the functional impacts of Gα_S_ mutations with the biophysical and structural properties of the variants measured in our experimental and computational data. Selected sites of mutation spanned four important structural regions. R201 is located in switch I; G226, Q227, and R228 are located in switch II; and R258 and R265 are located in switch III (Fig. 1). A366 is located between the β5 and α5 regions adjacent to the nucleotide-binding pocket, approximately 3.5 to 4.0 Å from the imidazole group of the guanine ring system.

Although the stability of Gα_S_ and the variants in the present study had not been previously directly measured *via* assays that monitor protein unfolding, a framework for predicting their relative stabilities was developed based on earlier structure-function studies. A crystal structure of GDP-bound Gα_S_[R201C] showed that this mutation altered an allosteric link between the nucleotide-binding pocket and the 3 Gα_S_ switch regions. The structure of GDP-bound Gα_S_[R201C] revealed an increased number of hydrogen bonds among the switch regions, which made GDP-bound Gα_S_[R201C] more closely resembled the structure of GTP-bound Gα_S_ (21). An increased number of hydrogen bonds were predicted to correlate with relatively higher stability for GTP-bound Gα_S_ and GDP-bound R201C, therefore correlating an increase in adenylyl cyclase activity for the R201C variant with an increase in the stability of GDP-bound R201C. The correlation between stability, hydrogen bonding networks, and aberrant function suggested a mechanism for the loss of adenylate cyclase activity for other Gα_S_ variants, in particular R228C, would involve an inverse effect. That is, destabilization of the GTP-bound conformation was predicted to correlate with loss-of-activity mutations (21).

Based on the above reasoning, we sought to investigate the extent to which changes in hydrogen bonding networks within the switch regions correlated with global protein stability and to also investigate the extent to which gain-of-function mutations in Gα_S_ shared the property of increased stability for their GDP-bound conformation. Concurrently, we also aimed to determine the extent to which a decrease of hydrogen bonding within the switch regions correlated with decreased global protein stability and to also determine the extent to which loss-of-function mutations shared the property of decreased stability for their GTP-bound conformation (Table 1). We predicted that comparing the stability and hydrogen bonding networks across a greater range of Gα_S_ variants would allow us to understand which variants shared similar mechanisms of gain of function with R201C or loss of function as R228C or whether alternative mechanisms were needed to explain the functional impact of other mutations.

**Table 1:** Properties and predicted impacts of Gαs disease-associated mutations

Mutation	Location in Gαs	Adenylyl cyclase activity	Predicted GDP-bound stability	Predicted GTP-bound stability	Predicted hydrogen bonding network
R201C	Switch l	Increased	Increased		Increased
Q227L	Switch ll loop	Increased	Increased		Increased
A366S	Connecting β6 and α5	Increased	Increased		Increased
G226A	Switch ll	Decreased		Decreased	Decreased
R228C	Switch ll	Decreased		Decreased	Decreased
R258A	Switch lll	Decreased		Decreased	Decreased
R265H	Switch lll	Decreased		Decreased	Decreased

Properties and predicted impacts of the studied mutations, including their location, previously reported impact on adenylyl cyclase activity, and the potential impacts on stability and hydrogen bonding network that were tested in this study. See also Table S1 for related properties of these variants.

Three of the seven studied mutations are documented to be gain-of-function mutations that increase cAMP accumulation: Gα_S_[R201C] (21), Gα_S_[Q227L] (22, 23), and Gα_S_[A366S] (4) (Table S1). For these, we sought to determine to what extent the mechanism proposed for the gain-of-function of Gα_S_[R201C] could be extended to the additional gain-of-function mutations. Four different loss-of-function mutations have been documented to result in decreased cAMP production: Gα_S_[G226A] (5), Gα_S_[R228C] (21), Gα_S_[R258A] (21) and Gα_S_[R265H] (21). For these variants, we sought to determine to what extent the mechanism proposed for Gα_S_[R228C] could be extended to explain loss of function for additional variants. For each of these variants, we next tested these predictions against experimental data measuring their stability (see below).

### Preparation of human Gα_S_ and Gα_S_ variants

**Figure 2:**
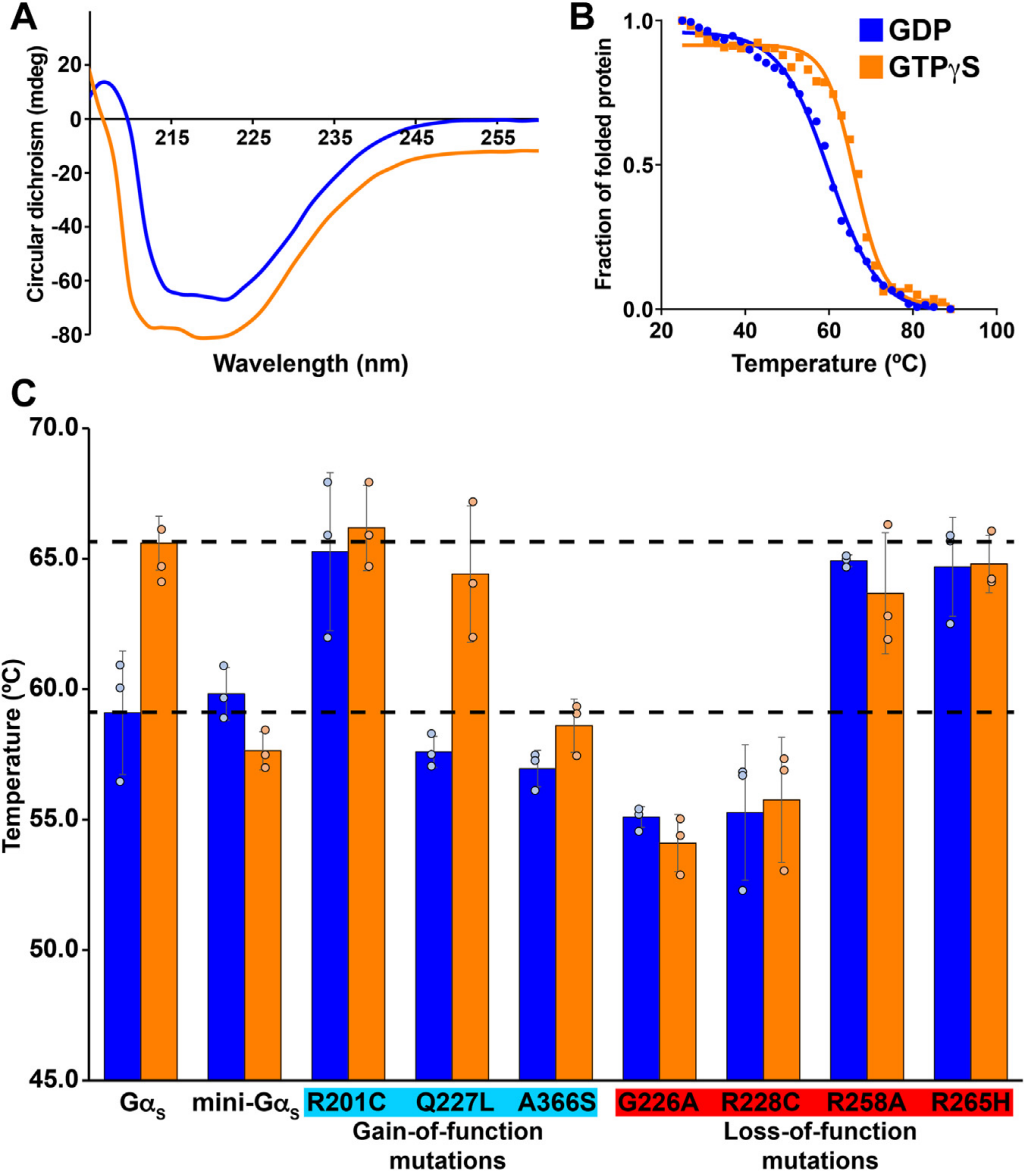
**Stability of GαS and GαS variants determined by variable-temperature CD spectroscopy.***A*, representative CD spectrum of GαS bound to GDP (*blue curve*) or GTPγS (*orange curve*). *B*, thermal unfolding of GαS bound to GDP or GTPγS monitored by variable temperature single wavelength CD spectroscopy. Same color scheme as *A*. *C*, histogram of Tm values determined from fitting the variable temperature CD data of GαS, mini-GαS, and GαS variants obtained in complex with GDP or GTPγS. Variant names are organized according to those with predicted increased stability for the complex with GDP (*cyan*) or predicted decreased stability for the complex with GTP (*red*), relative to GαS. *Dashed horizontal lines* are located at the mean Tm values for GαS in complexes with GDP or GTPγS. Error bars were determined from the SD of triplicate measurements.

Human Gα_S_ and all Gα_S_ variants were expressed in *Escherichia coli* and purified to >95% homogeneity using protocols adapted from earlier studies (21, 24); see Experimental procedures and Fig. S1, *A* and *B* for additional details. CD spectra of purified Gα_S_ and all Gα_S_ variants showed minima at 210 and 220 nm (Figs. 2*A* and S2), typical of folded proteins containing alpha helical secondary structure, confirming all samples were folded. For Gα_S_, structural rearrangements in the three switch regions associated with nucleotide exchange result in an increase in intrinsic tryptophan fluorescence (20). To verify protein function, we monitored changes in intrinsic tryptophan fluorescence upon exchange of GDP for the nonhydrolysable GTP analog GTPγS (Fig. S1). Rates of tryptophan fluorescence enhancement for Gα_S_ and Gα_S_ variants were consistent with values reported in prior studies (5, 21).

### Functional changes caused by mutations partially correlate with predicted changes in protein stability

Previous investigations documented that Gα_i1_ complexes with GTP or GTP analogs exhibited a ∼10 °C higher thermal denaturation temperature compared with GDP-bound Gα_i1_ (19, 25, 26). These observations were interpreted to indicate that Gα_i1_ adopted different conformations between the GDP and GTP-bound states, with the GTP-bound state exhibiting an increased number of hydrogen bonding among the switch regions giving rise to increased global structural stability (19, 25, 26).

To measure the stability of the 7 Gα_S_ variants and compare them with the stability of Gα_S_, we determined their thermal unfolding temperatures using variable temperature CD spectroscopy in the presence of either GDP or GTPγS. To provide an internal control, the study also included “mini-Gα_S_,” an engineered G protein with a truncation of the alpha helical domain and multiple mutations introduced to facilitate its crystallization in complex with GPCRs (24, 27). Mini-Gα_S_ recapitulates certain essential aspects of Gα_S_ binding to GPCRs, however it does not bind nucleotides with the same affinity as Gα_S_ and does not undergo global conformational rearrangements upon nucleotide exchange (24). The unfolding temperature (T_m_) of GTPγS-bound Gα_S_ was observed to be ∼9 °C higher than GDP-bound Gα_S_ (Fig. 2, *B* and *C*), consistent with earlier findings of Gα_i1_ (19, 25, 26).

Among the three variants with gain-of-increase mutations, we observed an increased T_m_ for GDP-bound Gα_S_[R201C], consistent with the earlier finding of an increased hydrogen bonding network that would result in increased stability (21). However, for both Gα_S_[Q227L] and Gα_S_[A366S] we observed a T_m_ value for their GDP-bound conformations to be similar to Gα_S_ (Fig. 2*C*), in contrast to an initial prediction of a higher T_m_. Interestingly, for Gα_S_[Q227L], we observed similar T_m_ values for both the GDP-bound and GTP-bound samples as compared with Gα_S_. For Gα_S_[A366S], we observed a lower T_m_ value for the GTP-bound protein, suggesting a global conformation for the GTP-bound protein that more closely resembles GDP-bound Gα_S_.

Among the four variants with loss-of-function mutations, we observed decreased T_m_ values for Gα_S_[R228C], consistent with the earlier prediction of decreased stability for the GTP-bound conformation of this variant (21). We also observed a decreased T_m_ value for Gα_S_[G226A], suggesting this variant may share a similar mechanism of loss of function (see Discussion). However, for both Gα_S_[R258A] and Gα_S_[R265H], we observed an increased T_m_ value for the GDP-bound conformation and a T_m_ value for their GTP-bound conformation similar to that of Gα_S_. Applying the same logic used to understand the functional and biophysical impact of the R201C mutation, the higher T_m_ value for GDP-bound Gα_S_[R258A] and Gα_S_[R265H] indicates an increased hydrogen bonding network for their GDP-bound conformations, in contrast to the prediction of a correlation between a loss of hydrogen bonding and decreased T_m_ value.

For comparison, we also recorded thermal unfolding data for mini-Gα_S_. As expected, we observed at most minimal differences in temperature between mini-Gα_S_ in the presence of GDP or GTPγS (Fig. 2), in line with the absence of structural differences observed in the presence of GDP or GTPγS and consistent with reports that claimed mini-Gα_S_ did not respond to the presence of GTP (24).

For the 3 Gα_S_ variants with increased T_m_ values of their GDP-bound conformations, Gα_S_[R201C], Gα_S_[R258A], and Gα_S_[R265H], we sought to understand what role the interaction with bound GDP played in providing increased stability by comparing the T_m_ values of these variants in the presence and absence of GDP. Gα_S_, Gα_S_[R201C], Gα_S_[R258A], and Gα_S_[R265H] were expressed and isolated without nucleotide added during the purification process. Purification of additional Gα_S_ variants without nucleotide was not attempted because these experiments focused on comparing Gα_S_ and variants that exhibited increased T_m_ values for their GDP-bound states. Apo Gα_S_ exhibited a T_m_ of ∼62 °C, higher than the T_m_ of GDP-bound Gα_S_ and lower than the T_m_ of GTP-bound Gα_S_ (Fig. S3). This suggests apo Gα_S_ adopts a global conformation that differs from both GDP-bound and GTP-bound Gα_S_. Interestingly, although Gα_S_[R201C], Gα_S_[R258A], and Gα_S_[R265H] all exhibited higher T_m_ values for complexes with GDP, we observed strikingly different behavior for apo preparations of these variants. For apo Gα_S_[R201C], we observed a lower T_m_ value of around 59 °C. Notably, both apo Gα_S_[R258A] and Gα_S_[R265H] exhibited higher T_m_ values similar to the T_m_ values in the presence of either nucleotide (Fig. S3). This indicated that the global conformations of Gα_S_[R258A] and Gα_S_[R265H] are similar both in the presence and absence of nucleotides but that Gα_S_[R201C] appears to adopt different conformations in the absence or presence of nucleotides. The implications for these observations on mechanisms of gain of function and loss of function are expanded on in the Discussion.

### MD simulations reveal changes in hydrogen bonding that correlate with thermal melting profiles of different Gα_S_ variants

Our framework for understanding the impact of the studied mutations posits that increased stability of Gα_S_ driven by mutations correlated with an increased hydrogen bond network. As discussed earlier, literature data of Gα_i1_ stability and structure showed a higher T_m_ value for GTP-bound Gα_I1_, which was attributed to increased interactions between switch I and switch II and between switch I and switch III upon complex formation of Gα_i1_ with GTP or GTP analogs (18, 19). The structure of GDP-bound Gα_S_[R201C] revealed an increased hydrogen bond network similar to that of GTP-bound Gα_S_ (21). Based on these earlier studies, we hypothesized that Gα_S_ variants with increased T_m_ values for complexes with GDP would exhibit an increased hydrogen bond network interactions, while Gα_S_ variants with decreased T_m_ values for complexes with GTP would exhibit a decreased hydrogen bond network interactions.

To investigate changes in the hydrogen bonding network, we conducted MD simulations on Gα_S_ and several Gα_S_ variants in complexes with GDP and GTP. We focused our efforts on simulating Gα_S,_ the gain-of-function mutation Gα_S_[Q227L], which showed unexpected thermal melting behavior similar to native Gα_S_, and the loss-of-function variants Gα_S_[R228C] and Gα_S_[R258A], which showed different thermal melting profiles from each other despite that both mutations cause loss of function.

**Figure 3:**
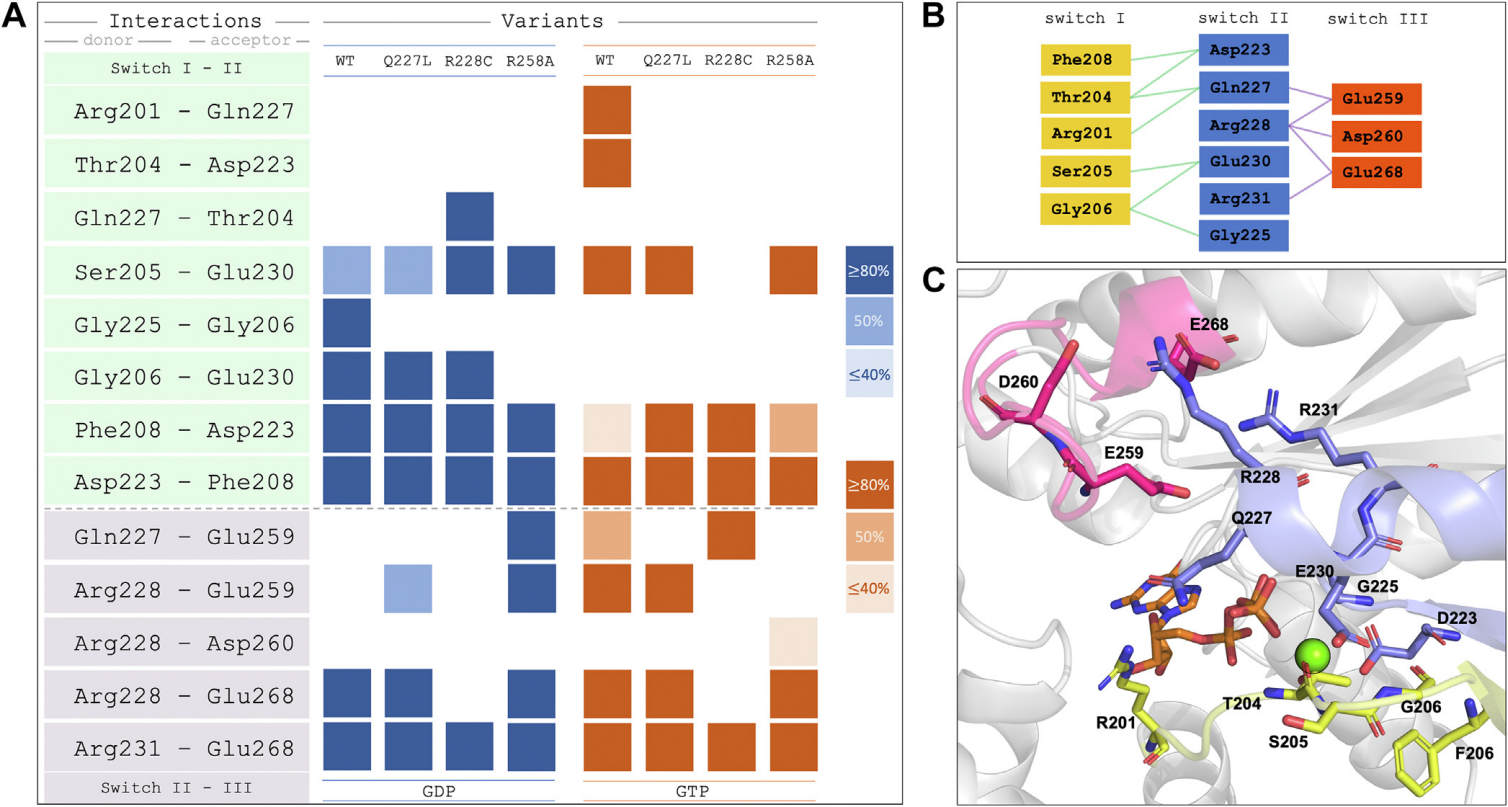
**Analysisof hydrogen bond interactions between switch regions extracted from MD simulations.***A*, hydrogen–bond interaction profiles for Gα_S_ (labeled “WT”), Gα_S_[Q227L], Gα_S_[R228C], and Gα_S_[R258A], focused on interactions between switch I, switch II, and switch III regions. *Colored boxes* indicate the existence of a persistent hydrogen bond, determined with different thresholds of occurrence along the given MD simulation, proportional to the color intensity for GDP-bound (*blue*) or GTP-bound (*orange*) proteins. *B*, schematic representation of all hydrogen–bond interactions depicted in the H-bond matrices. *C*, the structure of GTP-bound Gα_S_ with the involved residues from the analysis annotated. Residues from switch I are colored *yellow*, switch II colored *blue*, and switch III colored *red*. MD, molecular dynamics.

Based on our framework relating protein stability and hydrogen bonding interactions, we predicted that for the loss-of-function mutation Gα_S_[R228C] we should observe a decrease in the interactions within the hydrogen bonding network as compared with Gα_S_. Analysis of hydrogen bond interactions captured from MD simulations revealed that for Gα_S_[R228C] we observed significant loss of hydrogen bonds between switch I and switch II and between switch II and switch III for the GTP-bound state as compared with native Gα_S_ (Fig. 3). This is qualitatively consistent with the measured lower T_m_ value for GTP-bound Gα_S_[R228C] (Fig. 2). We observed the loss of an interaction between Ser205 in switch I and Glu230 in switch II for GTP-bound Gα_S_[R228C], and the loss of two additional interactions between switch II and switch III that were present for GTP-bound Gα_S,_ between Arg228 and Glu259 and between Arg228 and Glu268 (Fig. 3). Comparing all GTP bound proteins, we observed the fewest number of interactions between switch regions for Gα_S_[R228C].

In MD simulations of GTP-bound Gα_S_[R258A], we observed the hydrogen-bonding network to be more similar to that of native Gα_S_ than Gα_S_[R228C] (Fig. 3). As compared with Gα_S_[R228C], for Gα_S_[R258A] we observed several additional interactions between switch I and switch II and between switch II and switch III not observed for Gα_S_[R228C] (Fig. 3). While not identical to the hydrogen bond network observed for Gα_S_, this observation is qualitatively consistent with the similar T_m_ values measured for GTP-bound Gα_S_[R258A] and that of Gα_S_. For Gα_S_[R258A] bound to GDP, we also observed a notable increased hydrogen bond network, including increased hydrogen bond interactions between switch II and switch III and the presence of two interactions absent for Gα_S_, between Gln227 and Glu259 and between Arg228 and Glu259 (Fig. 3), which were absent for GDP-bound Gα_S_. This increased hydrogen bond network for GDP-bound Gα_S_[R258A] also appears qualitatively consistent with the increased T_m_ measured for GDP-bound Gα_S_[R258A] shown in Figure 2. Though both Gα_S_[R228C] and Gα_S_[R258A] are loss-of-function mutations, the distinct differences in their hydrogen bonding networks for both GDP-bound and GTP-bound conformations is consistent with their strikingly different stabilities and suggests two different mechanisms underlying their loss of activity (see Discussion).

MD simulations of Gα_S_[Q227L] showed patterns of hydrogen bonding interactions that were most similar to those of native Gα_S_ among the three variants for both GDP-bound and GTP-bound Gα_S_[Q227L] (Fig. 3). The only differences observed between Gα_S_[Q227L] and Gα_S_ were the loss of one interaction between switch II and switch III involving Q227 and E259 for the GTP-bound protein and the addition of one interaction between switch II and switch III for the GDP-bound protein. The similarities in hydrogen bond networks between Gα_S_ and Gα_S_ [Q227L] correlate with the differences in measured T_m_ values for Gα_S_[Q227L] bound to GDP and bound to GTPγS and the T_m_ values for Gα_S_ bound to GDP and bound to GTPγS (Fig. 2).

To complement the analysis of hydrogen bond networks, we also compared the dynamics of each switch region, described by the root mean square fluctuation (RMSF), among Gα_S_ and the Gα_S_ variants considered in this analysis. RMSF values were calculated from averaging over all MD trajectories for each variant and nucleotide combination. Fluctuations were determined as the average backbone deviation relative to the average position of the full trajectory per residue. We observed the largest RMSF for Gα_S_[R228C] for both the GDP and GTP-bound forms of this variant (Fig. S4), particularly around residues 225 to 230 of switch II. We also observed increased RMSF values across five residues of switch III for Gα_S_[R228C] (Fig. S4). This is in line with the decreased hydrogen bonding observed for residues between switch II and switch III of Gα_S_[R228A] (Fig. 3) and consistent with the observed lower T_m_ value for GDP-bound Gα_S_[R228C] shown in Figure 2.

Following methods for studying nonbonded interactions among switch regions in Gα_i1_ (19), the hydrogen bond analysis was further complemented with quantification of nonbonded interaction energies averaged over the MD trajectories, calculated for groups of residues defining the three switch regions (Fig. S5). The total interaction energy between groups of residues in switch I and switch II and between switch II and switch III were calculated and normalized with respect to GTP-bound Gα_S_. We observed a marked decrease in the nonbonded interaction energy for both GTP-bound and GDP-bound Gα_S_[R228C] (Fig. S5), consistent with analysis of the hydrogen bond network and decreased T_m_ value for GTP-bound Gα_S_[R228C]. Relative to Gα_S_[R228C], we observed an increase in the nonbonded interaction energy for both GTP-bound and GDP-bound Gα_S_[R258A] (Fig. S5). This is also consistent with our analysis of the hydrogen bond network for this variant and the increased T_m_ value for GDP-bound Gα_S_[R258A]. Interestingly, we did not observe decreased interaction energies for GDP-bound Gα_S_ or GDP-bound Gα_S_[Q227]. Thus, while quantification of interaction energies was consistent with observed T_m_ values for two of the variants, analysis of hydrogen bonding networks appears to be more qualitatively consistent with observed differences in protein stability.

### STD-NMR spectroscopy with Gα_S_ and Gα_S_ variants in complexes with nucleotides

From the allosteric model connecting the nucleotide-binding pocket to changes in interactions among the switch region, we hypothesized that the Gα_S_ disease-associated mutation should also alter interactions between nucleotides and nearby residues within the Gα_S_ nucleotide-binding pocket, even for sites of mutations that do not directly interact with nucleotides. We utilized STD-NMR spectroscopy to investigate the local structure and dynamics of residues within the nucleotide-binding pocket for the seven disease-associated variants. STD-NMR has been employed extensively to provide information on protein–ligand interactions and is especially suited for elucidating information with ligands that exhibit moderate binding affinities (K_d_ > 1 μM) (28, 29, 30, 31, 32). Well-isolated resonances from the protein, such as methyl groups that exhibit unique chemical shifts, are saturated in the ^1^H-NMR spectrum. If a ligand interacts with the protein, polarization is transferred to the ligand from the protein *via* spin diffusion (29). By subtracting the saturated spectrum from a reference spectrum, a difference spectrum is obtained where signals from protons of the interacting ligand are predominantly observed. The intensities of the observed ^1^H ligand signals depend on both the average distance and the dynamics of noncovalent interactions between the protein and ligand (29, 31).

STD-NMR experiments should be sensitive to changes in the local structure and dynamics within the nucleotide-binding pocket and provide complementary information to the CD measurements and MD simulations observing changes in interaction networks. For example, while the variable-temperature CD data and MD simulations did not reveal clear-cut differences between Gα_S_ and Gα_S_[Q227L], we hypothesized that local differences in the nucleotide-binding pocket may be observed by STD-NMR. Additionally, we were interested to explore whether STD-NMR data pointed to differences in potential mechanisms among the loss-of-function mutations Gα_S_[R258A], Gα_S_[R265H], and Gα_S_[R228C].

We recorded one-dimensional ^1^H STD-NMR data with Gα_S_ and the 7 Gα_S_ variants in the presence of a 50-fold excess of GDP or GppNHp (see Experimental procedures for more details). GppNHp was used rather than GTPγS due to different availabilities of the two compounds at the time of the study. Both GppNHp and GTPγS are widely used in studies of G proteins as both have been shown to exhibit affinities for most G proteins that differ by less than an order of magnitude (33, 34). ^1^H NMR signals for both GDP and GppNHp were identified and assigned in one-dimensional reference spectra (Fig. S7) by transferring assignments reported from earlier studies (Biological Magnetic Resonance Bank accession code bmse000270) (35). From exploratory STD-NMR experiments, we identified four ^1^H signals that were well resolved and free from residual protein background signals that were assigned to protons in the guanosine and sugar ribose groups, labeled as protons “a” through “d” (Fig. S7). STD-NMR experiments with GDP utilized signals from these four protons; in experiments with GppNHp, protons “c” and “d” were overlapped with background signal from the protein, and we therefore excluded them from analysis of samples containing GppNHp. Examination of the structure of Gα_S_ (PDB ID 1AZT) showed multiple residues within ∼6 Å of the protons monitored in STD-NMR experiments (Fig. S7). We anticipated these residues would make the largest contributions to observed STD-NMR signals and predicted that either structural rearrangement of these residues or their fluctuation relative to the bound nucleotide would result in changes in observed STD-NMR signals.

**Figure 4:**
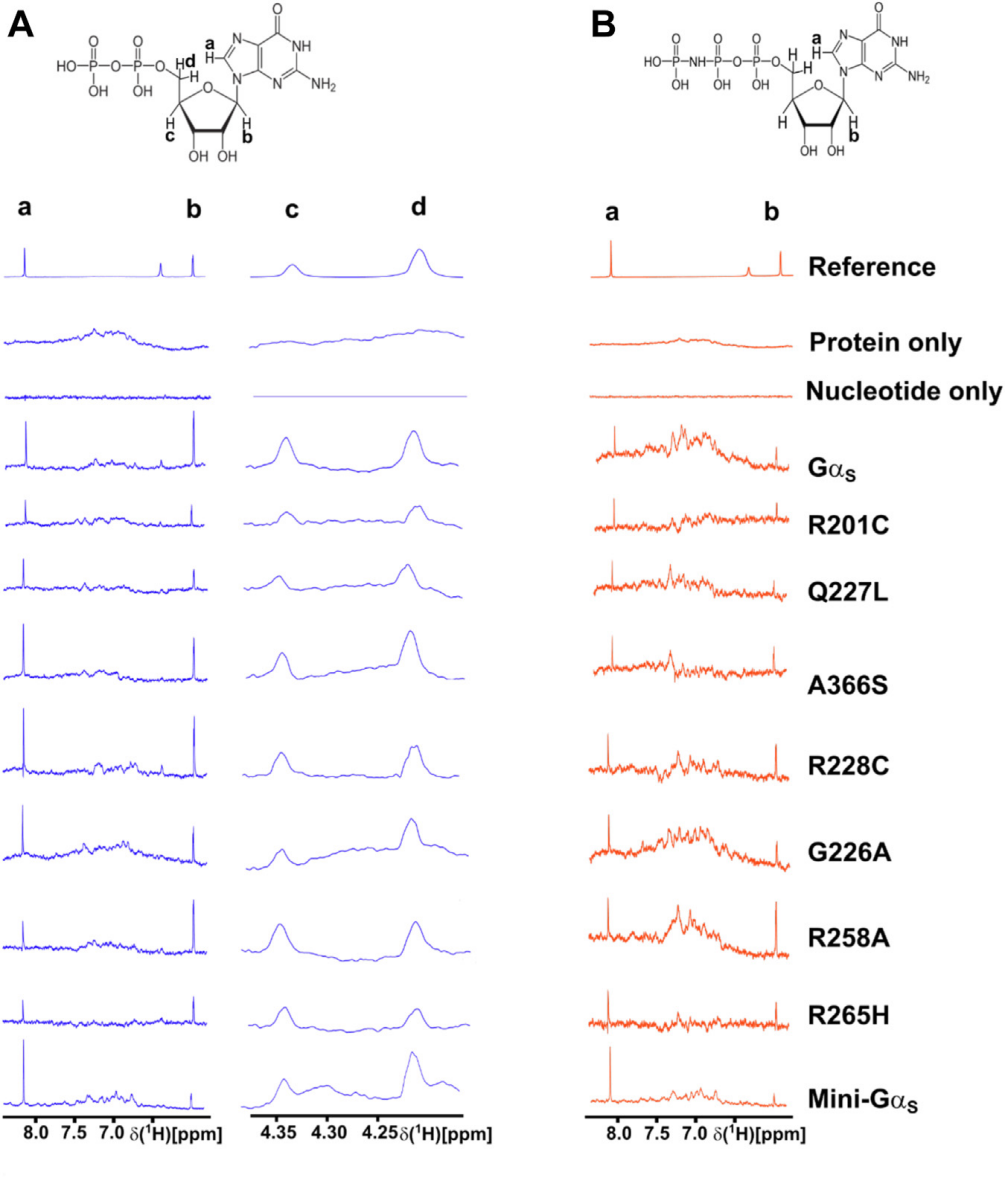
**One-dimensional**^**1**^**H STD-NMR spectra of Gα**_**S**_**and Gα**_**S**_**variants in complexes with GDP orGppNHp.***A*, the chemical structure of GDP and one-dimensional STD-NMR spectra of complexes with GDP. Protons observed in the STD-NMR spectra are annotated “a” to “d.” Below the chemical structure are expanded regions from one-dimensional STD-NMR spectra shown in Fig. S8 containing the annotated signals. “Reference” is a one-dimensional ^1^H NMR spectrum of GDP, “protein only” is an STD-NMR control experiment with a sample containing Gα_S_ and buffer but no nucleotide, and “nucleotide only” is a STD-NMR control experiment with a sample containing nucleotide and buffer but no protein. *B*, the chemical structure of GppNHp and one-dimensional STD-NMR spectra of complexes with GppNHp. Other presentation details are the same as in *A*. Views of the presented NMR data were expanded from the full spectra shown in Fig. S7. STD, saturation-transfer difference.

In initial STD-NMR experiments with Gα_S_ and GDP, we optimized the experimental signal-to-noise by varying the saturation transfer time from 0.5 to 3.0 s (Fig. S7). These data confirmed that interactions between nucleotides and Gα_S_ were detected in STD-NMR experiments and allowed optimization of the transfer time. We observed a significant increase in the STD-NMR signal up to 2.0 s, and only minor increases in STD-NMR signals were observed with a 3.0 s transfer time. We therefore selected 2.0 s for all subsequent experiments. Control experiments recorded for samples containing either only Gα_S_ and buffer or only nucleotide and buffer showed no STD-NMR signals, as expected (Fig. 4). This confirmed that signals observed in STD-NMR experiments were due to specific interactions between nucleotides and Gα_S_ or Gα_S_ variants.

The observation of STD-NMR signals for all variants provided important confirmation that nucleotides were bound to all variants. An earlier study of Gα_S_[R265H] reported that this variant did not bind GTP or GTP analogs (36), though this was later questioned (21). Importantly, we observed STD-NMR signals for Gα_S_[R265H], indicating that Gα_S_[R265H] does bind both GDP and GTP (Fig. 4) and is more in line with results by Hu and Shokat (21) (see Discussion). Detection of STD-NMR signals for all other Gα_S_ variants also confirmed their abilities to interact with and bind both GDP and the GTP analog.

In STD-NMR spectra of Gα_S_ or Gα_S_ variants with GDP, we observed significant variation in the signal intensities of protons “a” through “d” (Figs. 4 and S8). Mini-Gα_S_ showed the smallest signal intensities in STD-NMR spectra with respect to Gα_S_ for protons “b” through “d,” which appears consistent with its expected weaker nucleotide binding. The relatively largest signals for GDP were observed with the two variants Gα_S_[R258A] and Gα_S_[R265H] (Fig. 4). In STD-NMR spectra of Gα_S_ or Gα_S_ variants with GppNHp, we also observed a large variation in signal intensities, with mini-Gα_S_ again showing the weakest signal intensities (Figs. 4 and S8), confirming that the STD-NMR data were sensitive to the degree of interaction between the ligand and protein.

**Figure 5:**
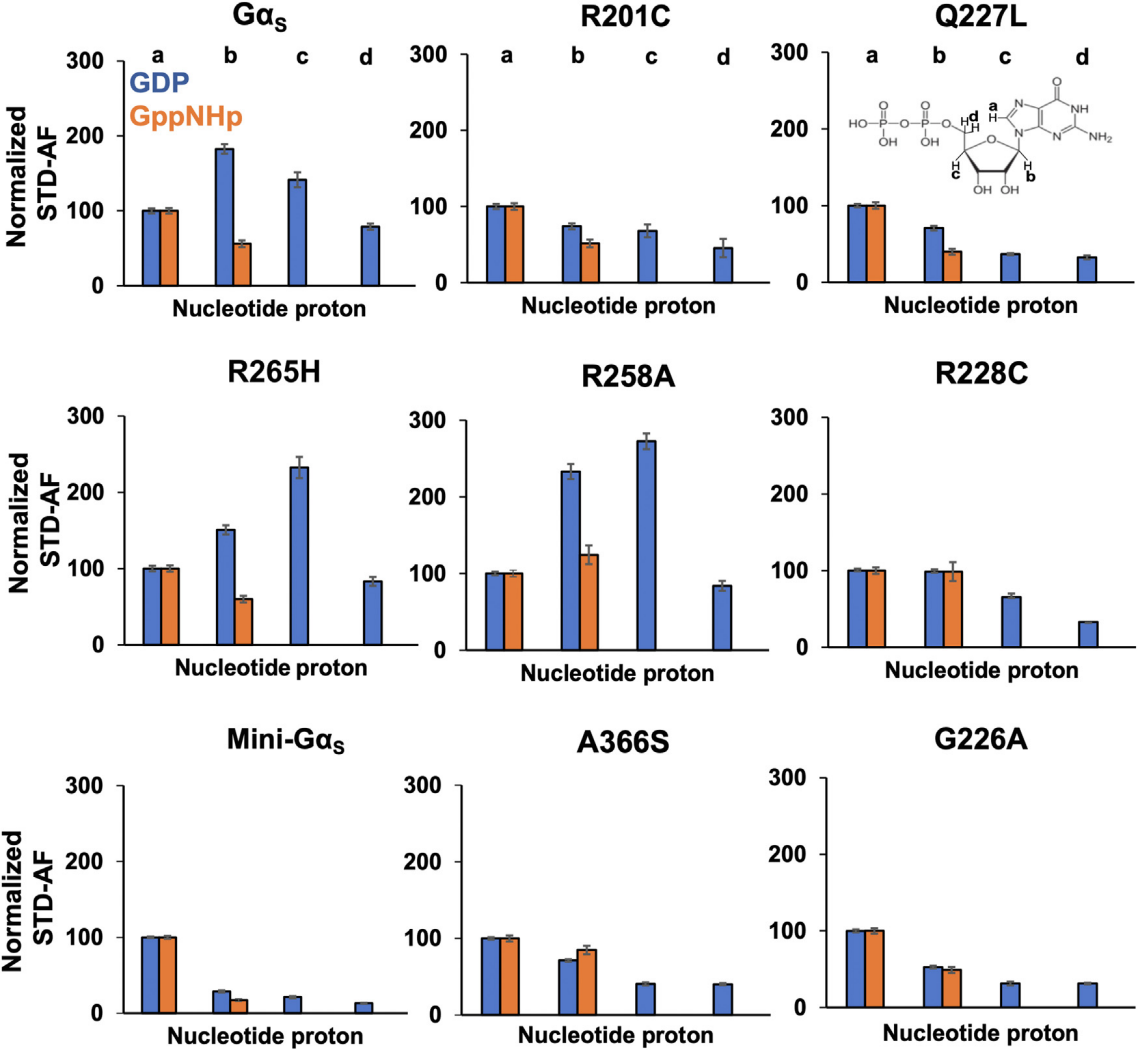
**Normalized STD amplification factors measured for GDP and GppNHp in complex with Gα**_**S**_**or Gα**_**S**_**variants.** Normalized STD amplification factors (STD-AF) determined for GDP (*blue*) and GppNHp (*orange*) in complex with Gα_S_ or Gα_S_ variants. The specific protons used to quantify the STD-AF values are annotated on the chemical structure of GDP. STD amplification factors were normalized to the “a” proton. Error bars were determined from the signal-to-noise ratios in the corresponding NMR spectrum. STD, saturation-transfer difference; AF, amplification factor.

We integrated the observed signal intensities from the STD-NMR spectra and used them to calculate corresponding STD amplification factors (STD-AFs) by multiplying the observed STD-NMR signal by the molar excess of ligand over protein (see Experimental procedures), following previously validated protocols (28, 29). The STD-AF converts the STD signal intensity, which depends on the fraction of bound ligand, to a value that depends on the fraction of bound protein (28, 29). We then calculated and compared the STD-AF values for all samples of Gα_S_ and Gα_S_ variants with GDP and GppNHp (Fig. 5). The STD-AF values were normalized with respect to the “a” proton of the guanine nucleobase, because in MD simulations, the immediate environment around the “a” proton showed little change (see below and Fig. S9).

**Figure 6:**
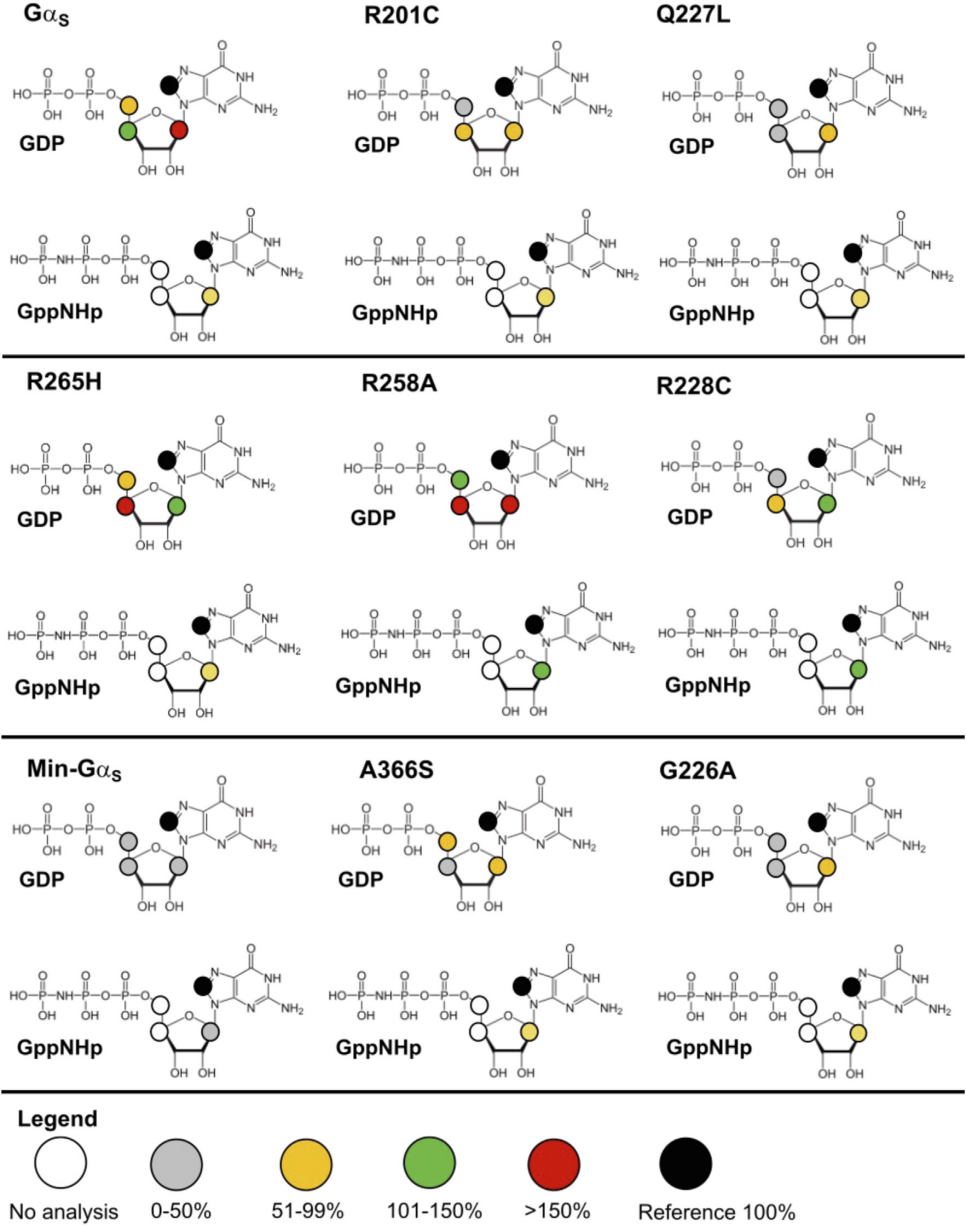
**STD-NMR amplification factors mapped onto the chemical structures of GDP and GppNHp for complexes with Gα**_**S**_**and Gα**_**S**_**variants.** For each proton observed in the STD-NMR spectra, the relative STD-AFs are colored as a percentage of the STD-AF normalized with respect to the “a” proton (same as in Figs. 3 and 4) indicated with the *black circle*. The STD-AFs are colored according to their relative intensities compared to those for the “a” proton: relatively weaker interactions representing <50% (*gray*), weak–moderate interactions representing 51% to 99% (*yellow*), moderate interactions representing 101% to 150% (*green*), and stronger interactions representing >150% (*red*). The *white circles* indicate that no analysis was performed for those protons due to overlap of nucleotide and background residual protein signals. AF, amplification factor; STD, saturation-transfer difference.

The normalized STD-AF values for samples of Gα_S_ or Gα_S_ variants prepared with either GDP or GTP provided a “fingerprint” of the extent of interaction of the nucleotide protons with local protons from the protein. For Gα_S_, relative to the “a” proton, we observed an increased STD-AF for the b and c protons with GDP, and a decrease in the STD-AF for the “b” proton with GppNHp (Figs. 5 and 6). We observed striking differences in the STD-AF profiles for both GDP-bound Gα_S_[R258A] and Gα_S_[R265H], as compared with Gα_S_ and all other Gα_S_ variants (Figs. 5 and 6). For both Gα_S_[R258A] and Gα_S_[R265H], we observed significant increases in their STD-AF values for complexes with GDP. This appears to qualitatively correlate with the rapid dissociation rate of GDP for these variants (36). For Gα_S_[Q227L], we observed significantly lower STD-NMR values than Gα_S_. This suggests that while the global conformations and switch region interaction networks are similar between Gα_S_[Q227L] and Gα_S_, the local structure and dynamics of residues within the nucleotide binding are different and reflected as differences in the STD-NMR data. We observed the smallest STD-AF values with GDP for mini-Gα_S_. Though the STD-AF values do not necessarily directly reflect binding affinities, the lower STD-AF values observed for mini-Gα_S_ are consistent with our expectations that this engineered protein exhibits significantly altered binding with nucleotides (24), which is also consistent a loss of interactions between bound nucleotides and residues nearby in both switch II and switch III regions removed to facilitate crystallization.

For protein complexes with GppNHp, STD-AF values calculated for “a” and “b” protons show a decrease in the value of the “b” proton relative to “a” for Gα_S_ (Figs. 5 and 6), establishing a “fingerprint” of interaction for the native protein. Compared with the STD-AF profile of Gα_S_, the STD-AF values for five of the Gα_S_ showed similar profiles, with two exceptions. Both Gα_S_[R258A] and Gα_S_[R228C] showed larger values observed for the “b” proton for their complexes with GppNHp (Figs. 5 and 6). While we did observe an STD-NMR signal for GppNHp in the presence of mini-Gα_S_, the value for the “b” proton was the lowest observed within the study, consistent with the expectation of altered binding of this engineered protein to the GTP analog (Figs. 5 and 6).

**Figure 7:**
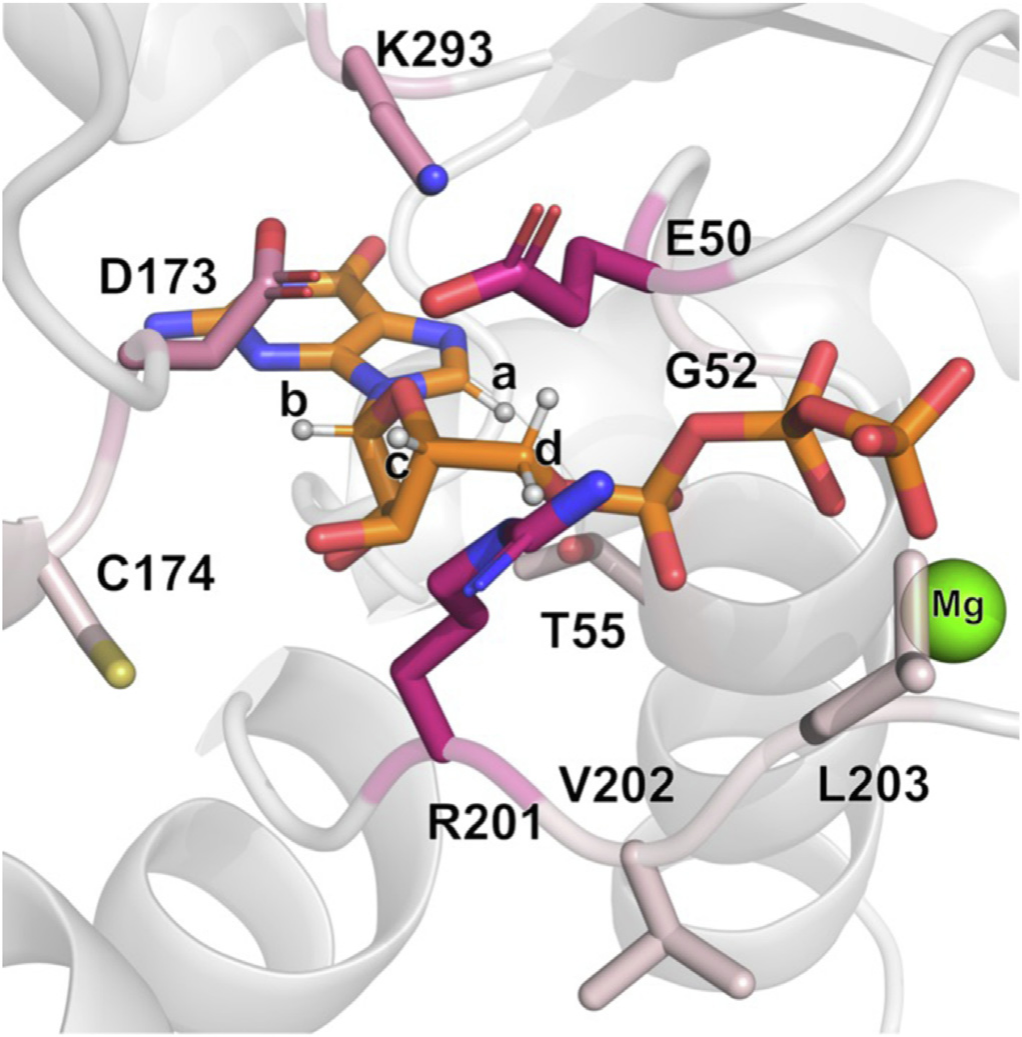
**Variation in the structure and dynamics of the nucleotide-binding pocket among Gα**_**S**_**and Gα**_**S**_**variants.** Nucleotide-binding pocket of Gα_S_ bound to GTP s(shown in *orange stick representation*, PDB:1AZT) with protons seen in STD–NMR interactions annotated “a” through “d.” The color palette for the residues interacting with these protons denotes increasing *shades of pink* which are proportional to the frequency of the residue as being the closest to the given proton, with *darker shades of pink* indicating higher frequencies as a function of the mutations explored. STD, saturation-transfer difference.

Observations from the STD-NMR data reflect changes in the local structure and dynamics of amino acids within the nucleotide-binding pocket of Gα_S_ and Gα_S_ variants; however, different configurations of the nucleotide binding region may result in similar STD-AF values. To obtain a more detailed view, we analyzed our MD simulations to investigate potential differences in the dynamics and configuration of the binding pocket, focusing on GDP-bound and GTP-bound Gα_S_ and the three variants explored in the earlier structural investigations underlying different thermal melting behavior, Gα_S_[R228C], Gα_S_[Q227L], and Gα_S_[R258A]. For each protein and for each nucleotide complex, we monitored each of the four protons on the nucleotide, “a” through “d,” observed in the STD-NMR experiments over the course of the simulations. In each simulation, we identified the closest protons in the protein within a radius of ∼6 Å of the nucleotide and then calculated the fraction of the simulation time that each residue spends in proximity to one or more of the monitored protons (Figs. 7, S9 and S10).

We observed both similarities and differences in the local environment among Gα_S_ and the 3 Gα_S_ variants. The “a” proton on the guanine ring of the nucleotide is in persistent proximity with G52 for both GDP-bound and GTP-bound Gα_S_ and for all variants, with an overall mean occupancy across all proteins of 97.4 ± 1.4% (Figs. 7 and S9). The associated SEM was used as an indicator of the variability, which was relatively low in this case. As discussed above, this mostly uniform local environment around proton “a” rationalized our normalization of the STD-AF values with respect to its signal intensity (Figs. 5 and 6).

The “b” proton is mainly interacting with Asp173 (mean occupancy of 67 ± 14%) and Lys293 (32.2 ± 14.1%, Fig. S9). Most of this variability is due to the significant difference between the GDP and GTP-bound forms in both Gα_S_[Q227L] and Gα_S_[R258A] mutants, where the GTP-bound form shows an even contact between the “b” proton and Asp173 and Lys293. Interestingly, the STD-AF normalized values of these two mutants show a gap for the “b” proton interactions between their corresponding GDP and GTP complexes. For the remaining protons, we were unable to experimentally measure their GTP-bound normalized STD-AF values. Nevertheless, we can examine results from MD simulations to make predictions on their behavior. Proton “c” exhibits the highest variability in the identity of the closest residue among the different Gα_S_ forms with shared interactions with Arg201, Asp173, and Glu50; however, for Gα_S_[Q227L], proton “c” almost exclusively interacts with Glu50 when bound to GDP or with Arg201 when bound to GTP. Analysis of the “d” protons in GDP was more complex since this signal is due to two equivalent protons. To address this, we analyzed the arithmetic average of the independent measurements for each proton, and the results show a constant and even distribution of the closest interacting residues, with Glu50 and Arg201 making an even interaction with the “d” protons in all simulations. Moreover, in all four protein systems (Gα_S_ or variants Gα_S_[Q227L], Gα_S_[R258A], and Gα_S_[R228C]) the GTP-bound form displays a distinct interaction of the “d” protons with Gly52 (average occupancy 13.0 ± 3.8%, *versus* 0.6 ± 0.8% in the corresponding GDP-bound forms, Fig. S9), suggesting a different microenvironment of these protons as a function of the nucleotide bound.

## Discussion

Based on the reported crystal structure of Gα_S_[R201C] and biochemical data of Gα_S_[R228C], mechanisms for Gα_S_ gain-of-function and loss-of-function mutations were proposed that related changes in hydrogen bond networks within the switch regions to changes in protein stability and function (21). This framework was built upon earlier biophysical and biochemical experiments with Gα_i1_ that related changes in Gα_i1_ thermal stability to changes in the hydrogen bond network among switch region residues, which were also closely related to global structural rearrangements and changes in Gα_i1_ function (18, 19, 26). We leveraged an integrative biophysical approach to investigate the extent to which this framework could be expanded to provide mechanistic insights into additional disease-associated Gα_S_ variants.

With this biophysical toolbox, we examined four loss-of-function Gα_S_ variants: Gα_S_[G226A], Gα_S_[R228C], Gα_S_[R258H], and Gα_S_[R265H]. Based on biochemical data, it had been proposed that the R228C mutation resulted in loss of function due to destabilization of its GTP-bound conformation (21). We observed a decreased T_m_ for GTP-bound Gα_S_[R228C] with respect to GTP-bound Gα_S_, indicating decreased stability (Fig. 2), and we correlated this observation with the loss of hydrogen bonds in the switch region network for this variant (Fig. 3). We also observed the largest RMSFs within the switch region for GTP-bound Gα_S_[R228C] (Fig. S4). Taken together, these data are consistent with the explanation of a destabilized GTP-bound state for this variant. Our observations of decreased stability for GTP-bound Gα_S_[G226A], closely similar to Gα_S_[R228C], suggest that the same mechanism likely explains the impact of the G226A variant as well.

For the loss-of-function variants Gα_S_[R258A] and Gα_S_[R265H], we observed the stability for these GTP-bound variants to be similar to that of Gα_S_, in contrast to Gα_S_[G226A] and Gα_S_[R228C] (Fig. 2). For Gα_S_[R258A] and Gα_S_[R265H], we also observed increased stability for their GDP-bound states, which was found to be consistent with increased hydrogen bond networks in MD simulations (Figs. 2 and 3). While our observations for Gα_S_[G226A] and Gα_S_[R228C] confirmed the correlation between increased stability and increased interactions within the switch regions, this was not consistent with the behavior of other loss-of-function variants Gα_S_[R228C] and Gα_S_[G226A], indicating that an alternative mechanism must account for their loss of function.

Interestingly, for nucleotide free Gα_S_[R258A] and Gα_S_[R265H], we observed T_m_ values that were similar to the higher T_m_ value observed for GTP-bound Gα_S_ (Fig. S3). The increased stability of nucleotide-free Gα_S_[R258A] and Gα_S_[R265H] indicates that these two variants adopt stable, relatively rigid states in the absence of any nucleotide. This appears to be a unique property of these variants and is not shared with nucleotide free Gα_S_ or Gα_S_[R201C]. Based on these data, we propose an alternative mechanism to explain the loss of function in Gα_S_[R258A] and Gα_S_[R265H]. Specifically, we propose that Gα_S_[R258A] and Gα_S_[R265H] are “locked” in a conformation that is suboptimal for binding GDP or GTP, impairing the protein's ability to undergo conformational changes that support stronger interactions with nucleotides, resulting in rapid dissociation of GDP and rapid hydrolysis of GTP. This alternative mechanism is supported by earlier studies that reported Gα_S_[R265H] did not bind GTP (36). Leyme *et al.* based their conclusion that Gα_S_[R265H] did not bind GTP based on the lack of increased inherent tryptophan fluorescence upon the addition of GTP (36). However, more recent studies by Hu and Shokat, using different approaches that did not rely on inherent tryptophan fluorescence, concluded that Gα_S_[R265H] does bind GTP (21). From the STD-NMR data in the current work, we observed that Gα_S_[R265H] and Gα_S_[R258A] bind both GDP and GTP analogs (Figure 4, Figure 5, Figure 6). This discrepancy with Leyme *et al.*'s conclusion can be explained by the idea that Gα_S_[R265H] does not undergo conformational changes upon nucleotide exchange that would affect the environment around tryptophan residues, particularly W234, which is adjacent to the switch regions, leading to no change in tryptophan fluorescence intensity. One implication from this idea is that the rigidity of Gα_S_[R265H] and Gα_S_[R258A], even in the nucleotide-free state, suggests these variants may be less suitable as targets for potential small molecule therapeutics that require access to flexible binding pockets.

For the gain-of-function mutations Gα_S_[R201C], Gα_S_[Q227L], and Gα_S_[A366S], based on the earlier crystal structure and mechanistic study of GDP-bound Gα_S_[R201C] (21), we wanted to first test to what extent their relative stabilities correlated with changes in hydrogen bonding networks, and, second, to what extent these observations could be correlated with their changes in functional activity. For Gα_S_[R201C], we observed an increased stability and increased hydrogen bond network (Figs. 2 and 3) for GDP-bound Gα_S_[R201C]. This is consistent with the earlier claim of increased stability for this variant based on the crystal structure (21). For Gα_S_[A366S], we observed a lower T_m_ for the GTP-bound conformation, indicating a loss of stability and likely loss of hydrogen bonding network for GTP-bound Gα_S_[A366S], destabilizing the GTP-bound conformation of this variant (Figs. 2 and 3). Earlier measurements of Gα_S_[A366S] nucleotide binding kinetics showed a decreased ability of this variant to bind GDP (37), suggesting a plausible contribution toward its increase in function. Our analysis indicates that another contributing factor toward increased function is the destabilization of the GTP-bound conformation. Though this observation differs from the increased stability observed for GDP-bound Gα_S_[R201C], it is line with earlier studies of A326S in Gα_i_ that showed this mutation altered the conformation of the nucleotide-binding pocket through steric crowding (38), which is consistent with our observations of a destabilized GTP-bound conformation.

Surprisingly, Gα_S_[Q227L] is the only variant that exhibited stability for both GDP and GTPγS complexes highly similar to native Gα_S_ (Fig. 2). This was the only variant that did not exhibit a change in stability for either the GDP-bound or GTP-bound states. Consistent with this result, we also observed similar hydrogen bonding networks between Gα_S_[Q227L] and Gα_S_ (Fig. 3), indicating that this particular mutation does not inhibit this variant’s ability to adopt different global conformations for complexes with different nucleotides. Several unique features were observed in MD simulations for Gα_S_[Q227L] (Figs. 7 and S9), providing potential clues to its gain in function. For Gα_S_[Q227L], proton “c,” located on the pentose sugar ribose of GDP, exhibited significant differences in its local environment as compared with Gα_S_, Gα_S_[R228C], and Gα_S_[R258A]. Within the local environment of proton “c,” for Gα_S_[Q227L], we observed a significant increase in the percent contact with residue Glu50 (96.3%) and loss of contact with residues Asp173 and Arg201. While these differences are more subtle than for other variants, they may partially contribute toward functional differences and reflect differences in the dynamics of residues within the nucleotide-binding pocket between Gα_S_[Q227L] and Gα_S_.

In summary, from our combined biophysical and computational data, we observed a clear correlation between protein stability and changes in hydrogen bonding within the switch region for a series of seven disease-associated Gα_S_ variants containing single amino acid mutations. Six of the seven variants could be identified and categorized based on observations of their stabilities, suggesting that experiments measuring the stability of additional Gα variants may be sufficient for providing a plausible mechanism of loss or gain of activity. Comparison of the relative stabilities of these variants for complexes with nucleotides, the hydrogen bonding network within the switch regions, and structural dynamics within the nucleotide-binding pocket provided evidence supporting five mechanisms that give rise to changes in function. Four of these mechanisms are related by the observation that changes in stability for either GDP-bound or GTP-bound variants correlate with changes in hydrogen bond networks and can rationalize protein function. In the first two related mechanisms, Gα_S_[R201C] results in a gain of function by stabilization of the GDP-bound conformation while Gα_S_[R228C] and Gα_S_[G226A] result in loss of function by destabilization of their GTP-bound conformations. In a third mechanism, Gα_S_[R265H] and Gα_S_[R258A] result in a loss of function by being locked into conformations that are not optimal for binding GDP and that also result in rapid GTP hydrolysis. In a fourth mechanism, Gα_S_[A366S] results in destabilization of the GTP-bound conformation, possibly due to steric crowding around the nucleotide-binding pocket. Evidence for these four mechanisms could be observed from the combined CD and NMR data and MD simulations relating changes in variant stability with changes in hydrogen bond networks and function. The variant Gα_S_[Q227L] was the only one that could not be readily distinguished from the stability or hydrogen bonding interactions observed for Gα_S_, suggesting a more subtle mechanism underlies its activity. We anticipate that by making similar measurements with additional Gα variants, future research may be able to categorize mechanisms of loss or gain of function for many other variants based on similar biophysical data.

## Experimental procedures

### Generation of Gα_S_ and variant Gα_S_ plasmids

An initial Gα_S_ construct was gifted from the laboratory of Prof. Kevan Shokat (UCSF) (21), contained in a pET15b vector and derived from the short human isoform of Gα_S_ (PubMed accession number NP_536351) with the first seven residues deleted. We modified this plasmid by replacing a DRICE cleavage site with a tobacco etch virus nuclear-inclusion-a endopeptidase (TEV protease) cleavage site. This plasmid was used to express Gα_S_ for our experiments. All Gα_S_ variants were obtained from this plasmid by site-directed mutagenesis using the QuikChange II kit (Agilent) and primers listed in Table S2. The plasmid encoding for mini-Gα_S_ was obtained from GenScript and designed to be identical to the sequence of the mini-Gα_S_ “393” sequence (24).

### Expression and purification of GαS and GαS variants

Each Gα_S_ plasmid was transformed into BL21-CodonPlus (DE3)-RIL cells (Agilent), and the bacteria were grown at 30 °C in M9 medium (50 mM Na_2_HPO_4_, 25 mM KH_2_PO_4_, 8.5 mM NaCl, 1 mM MgSO_4_, 50 μM FeCl_3_, 100 μM CaCl_2_, 44 mM glucose, 56 mM NH_4_Cl, and 0.01% w/v thiamine) with 34 μg/ml chloramphenicol and 100 μg/ml carbenicillin. Cells were grown to an absorbance (*A*_600_ nm) between 0.6 and 0.8 and protein expression was then induced with 50 μM IPTG at 25 °C for 18 to 20 h. Cells were sedimented by centrifugation at 4000*g* at 4 °C for 20 min then were lysed using a cell disruptor at 24,000 psi and 4 °C in buffer containing 50 ml of 25 mM Tris pH 8.0, 150 mM NaCl, 1 mM MgCl_2_, 50 μM GDP with EDTA-free in-house protease inhibitor cocktail (500 μM 4-(2-aminoethyl)-benzenesulfonylfluoride hydrochloride, 1 μM E−64, 1 μM leupeptin, 150 nM aprotinin) at a ratio of 50 ml lysis buffer for every 1 l of cell culture. The lysate was clarified by centrifugation at 25,000 rpm at 10 °C. The lysate was incubated overnight (16–20 h) with 3 ml of nickel-nitrilotriacetic acid resin (Cytiva) for every 1 l of cell culture at 4 °C. Then, the resin was washed two times with 50 ml wash buffer containing 25 mM Tris pH 8.0, 500 mM NaCl, 1 mM MgCl_2_, 5 mM imidazole, and 50 μM GDP at 4 °C. The first wash included 2 mg/ml iodoacetamide. The protein was eluted at 4 °C in a gravity flow column using a buffer containing 25 mM Tris pH 8.0, 250 mM NaCl, 1 mM MgCl_2_, 250 mM imidazole, 10% vol/vol glycerol, and 50 μM GDP. The protein was then exchanged into a buffer containing 5 mM Tris pH 8.0, 100 mM NaCl, 10% vol/vol glycerol, 1 mM MgCl_2_, and 50 μM GDP *via* an Akta Start FPLC equipped with a HiPrep 26/10 desalting column (Cytiva). The protein was then incubated overnight at 4 °C with thermostabilized TEV protease (39) at a 1:50 M ratio (Gα_S_: TEV). After overnight incubation (16–20 h), cleaved Gα_S_ was purified *via* reverse immobilized mobile affinity chromatography by incubating the sample with nickel affinity resin at 4 °C for 30 min and collecting the flow through using a gravity flow column. A final purification step was performed by size-exclusion chromatography *via* an Akta Pure FPLC equipped with a Superdex 200 increase 10/300 Gl column (Cytiva) equilibrated in buffer containing 20 mM Hepes pH 8.0, 150 mM NaCl, 5 mM MgCl_2_, 1 mM EDTA, 10% v/v glycerol, and 10 μM GDP. Peak fractions containing purified Gα_S_ or Gα_S_ variants were isolated and pooled for subsequent experiments. Ten percent SDS gels were prepared to check the purity and confirm the identity of the protein. The gel was made with running buffer consisting of 1 M Tris pH 8.4, 10% acrylamide (Bio-Rad), 0.1% SDS, 0.12% ammonium persulfate, 8 mM tetramethylethylenediamine (Bio-Rad), and stacking buffer consisting of 0.75 M Tris pH 8.4, 5% acrylamide (Bio-Rad), 0.1% SDS, 0.1% ammonium persulfate, 7 mM tetramethylethylenediamine (Bio-Rad), and run from 10X stock of cathode buffer (1 M Tris pH 8.4, 1M tricine, 1% SDS) and 10X anode buffer (2 M Tris pH 8.9) diluted to 1X. Apo Gα_S_ proteins and variants (without nucleotide) were purified in the same manner as above but without GDP in any of the buffers.

### Expression and purification of mini-GαS

We expressed and purified mini-Gα_S_ using a strategy adapted from earlier studies (24, 27). BL21-CodonPlus (DE3)-RIL were transformed with the expression vectors and grown at 30 °C in Terrific broth medium with 0.2% glucose, 34 μg/ml chloramphenicol, 100 μg/ml carbenicillin, and 5 mM MgSO_4_. Protein expression was induced with 50 μM IPTG at 25 °C and left to grow overnight (16–20 h). Cells were lysed in 40 mM Hepes pH 7.5, 100 mM NaCl, 10 mM imidazole, 10% vol/vol glycerol, 5 mM MgCl_2_, 50 μM GDP with EDTA free in-house protease inhibitor cocktail (500 μM 4-(2-aminoethyl)-benzenesulfonylfluoride hydrochloride, 1 μM E-64, 1 μM leupeptin, 150 nM aprotinin), and using a cell disruptor operating at 24 kPsi and 4 °C. The lysate was clarified by centrifugation at 25,000 rpm and 10 °C for 30 min and loaded on a His-Trap HP NiNTA 5 ml column (Cytiva) *via* an Akta Start FPLC. Resin was washed with lysate buffer containing a final concentration of 40 mM imidazole and 500 mM NaCl, and protein was eluted from the resin with lysate buffer containing 400 mM imidazole. The sample was exchanged into buffer *via* an Akta Start FPLC equipped with a HiPrep 26/10 desalting column (Cytiva) that was equilibrated in 20 mM Hepes pH 7.5, 100 mM NaCl, 10% vol/vol glycerol, 1 mM MgCl_2_, and 10 μM GDP. Fractions containing mini-Gα_S_ were collected and incubated for 16 to 20 h at 4 °C with thermostabilized TEV protease at 1:100 M ratio (mini-Gα_S_:TEV). Cleaved mini-Gα_S_ was purified *via* reverse immobilized mobile affinity chromatography by incubating the sample with nickel resin for 30 min and collecting the flow through using a gravity flow column. Mini-Gα_S_ was further purified *via* an Akta Start FPLC equipped with a HiLoad Superdex 75 pg column (Cytiva) equilibrated in storage buffer (10 mM Hepes pH 7.5, 100 mM NaCl, 10% vol/vol glycerol, 1 mM MgCl_2_, 1 μM GDP, and 0.1 mM tris(2-carboxyethyl)phosphine). Purified samples were rapidly frozen in liquid nitrogen and stored at −80 °C until needed for experiments.

### Tryptophan fluorescence assays

Gα_S_ and Gα_S_ variants were exchanged into buffer containing 20 mM Hepes pH 8.0, 150 mM NaCl, and 5 μM GDP using a PD MiniTrap G25 column (Cytiva), and the sample volumes were adjusted to achieve a final concentration of 10 μM protein. Tryptophan fluorescence spectra were recorded with a Cary Eclipse spectrometer operating at 25 °C. The excitation wavelength was 280 nm and emission was observed at 340 nm with 5 nm excitation and emission bandwidths. GTPγS was added to 200 μl protein for a final concentration of 50 μM in a quartz cuvette. The sample was mixed vigorously by repeated pipetting and data collection immediately started. Data points were recorded every 12 s for 200 min total acquisition time. Data were analyzed in GraphPad prism (https://www.graphpad.com) using the one-phase association equation shown below:

$$Y=Y_{O}\;+\;\left(Plateau\;-\;Y_{o}\right)*\left(1\;-\;e^{\left(-kx\right)}\right)$$

where $Y_{o}$ is the fluorescence intensity when *x* (time) equals zero, “Plateau” is the fluorescence intensity when the system has been fully equilibrated at infinite time expressed in the same units as Y, and k is the rate constant, expressed as the reciprocal of the *x*-axis time units.

### Protein thermal unfolding monitored by CD

Single-wavelength variable temperature CD data were recorded on a Chirascan qCD spectrometer. Gα_S_ or Gα_S_ variants were concentrated to 10 μM in buffer containing 10 mM Hepes pH 7.5, 100 mM NaCl, 1 mM MgCl_2_, and 50 μM GDP or 50 μM GTPγS. First, full-wavelength CD spectra were recorded at 25 °C to confirm samples were properly folded. Then, protein melting was monitored by recording the ellipticity at 220 nm from 25 °C to 89 °C with a linear heating rate of 1 °C/min. Each T_m_ was determined by fitting the data in GraphPad prism to a Boltzmann sigmoidal equation shown below:

$$Y\;=\;\text{Bottom}\;+\;\frac{\left(\text{Top}\;-\;\text{Bottom}\right)\;}{1\;+\;\exp(\frac{V50\;-\;X)}{\text{Slope}})}$$

where Y is the fraction of folded protein as a function of temperature, V_50_ is the temperature at which half of the protein is unfolded, X represents the temperature, and “slope” is the steepness of the curve. “Top” and ‘Bottom’ are normalization factors set to 1.0 and 0, respectively. Error bars were determined by calculating the standard deviation from three independent measurements.

### NMR experiments

Samples of Gα_S_ or Gα_S_ variants were exchanged into NMR buffer using a PD MiniTrap G25 column (Cytiva) equilibrated with 25 mM HEPES pH 7.0, 75 mM NaCl, 5 mM MgCl_2_, 100 μM DSS, 10% v/v ^2^H_2_O, and either 2 mM GDP or 2 mM GppNHp. Exploratory experiments employing a range of protein-to-ligand concentrations were tested to determine optimal STD-NMR signal-to-noise. Based on these data we prepared samples containing 40 μM protein and 2 mM ligands (Fig. S6). NMR experiments were recorded at 25 °C and 800 MHz ^1^H Larmor frequency using a Bruker Avance III spectrometer equipped with a 5 mm TXI cryoprobe. One-dimensional-^1^H saturation transfer difference (STD) experiments were recorded using water suppression by gradient-based excitation sculpting (Bruker pulse program stddiffesgp), with 64 scans per experiment and a 3 s delay between scans. On-resonance spectra were acquired by irradiating at 0.79 ppm to target Gα_S_ methyl protons, and off-resonance spectra were acquired by irradiating at −40.0 ppm. Saturation times were tested from 0.5 to 3 s, and an optimal saturation time of 2 s was determined (Fig. S8). Data were acquired and analyzed in Topspin 4.1.1. Prior to Fourier transformation, the data set was multiplied by an exponential window function applying 3 Hz line broadening and zero-filled to 65,536 points; baseline correction was applied to all frequency domain data sets. To reduce background signals, a reference STD-NMR spectrum was recorded with a sample containing the same concentration of protein but no nucleotide. This spectrum was processed identically and subtracted from all STD-NMR spectra.

AFs, $A_{\text{STD}}$, were determined from the integrals of ligand signals in the STD difference ($I_{0}-\;I_{\text{sat}}$) and reference ($I_{0}$) spectra according to the following equation from reference (29)

$$A_{\text{STD}}\;=\;\frac{I_{0}\;-\;I_{\text{sat}}}{I_{0}}\;\times \;\frac{\left[L\right]}{\left[P\right]}$$

where $A_{\text{STD}}$ is the AF of each STD signal, $I_{O}$ is the integral of the STD signal in the reference spectrum, $I_{\text{sat}}$ is the integral of the STD signal in the difference spectrum and [L] and [P] are the concentrations of ligand and protein, respectively.

Signal-to-noise ratios in the STD difference spectra, ${SNR}_{diff}$, were determined for each ligand signal using the “sinocal” function on topspin 4.1.1. Error associated with the AFs, σ ,was evaluated using the following equation:

$$\sigma \;=\;A_{\text{STD}}\;\times \;\frac{1}{{\text{SNR}}_{\text{diff}}}$$

where $A_{\text{STD}}$ is the AF of each STD signal and ${\text{SNR}}_{\text{diff}}$ is the signal-to-noise ratio of the STD signal. For presentation of the normalized $A_{\text{STD}}$ signals, each nucleotide signal “a” to “d” and associated errors were normalized by the $A_{STD}$ of the “a” signal.

### MD simulations

The crystal structure of the Gα_S_ protein in complex with GTPγS was retrieved from the Protein Data Bank (PDB ID 1AZT) (40) and prepared for MD simulations as follows. Chain B from the dimer in the asymmetric unit was retained, and the missing segment between α1 and αA (residues 70–86) were modeled with Prime (Schrӧdinger Release 2011–2: Prime; Schrӧdinger, LLC: New York, 2011). Crystallographic water molecules beyond 5 Å of GTPγS as well as all cocrystallized phosphate ions were removed, and the protonation states of ionizable residues were determined with PROPKA at pH 7.0. The GTPγS substrate was modified to either GTP or GDP, depending on the intended system, with Maestro v 9.4 (Schrӧdinger, LLC). Amino acid replacements to generate the different Gα_S_ variants were produced with the Schrödinger’s PyMOL mutagenesis tool (The PyMOL Molecular Graphics System, Version 2.0. Schrӧdinger, LLC.).

MD simulations were executed with the Q software package (41), using the OPLS-AA/M force field for proteins and the TIP3 water model (42), available from https://github.com/qusers/q6. The parameters to describe GTP and GDP were taken from same force field. All models were partially solvated in a water sphere of diameter 50 Å with its center located at the nucleotide’s center of geometry. Protein atoms outside the sphere were tightly constrained to their initial coordinates with a force constant of 200 kcal mol^−1^ Å^−1^ and excluded from nonbonded interactions, while water molecules at the sphere boundary were subjected to radial and polarization restrains following the SCAAS model (43). All atoms inside the simulation sphere were allowed to move freely, where the local reaction field multipole expansion was used for long-range electrostatic interactions beyond a cut-off of 10 Å (44). Each system was subject to 100 independent replica MD simulations, each of them starting with an equilibration phase consisting of 51 ps of gradual heating to 298 K and concurrent release of (initial 10.0 kcal mol^−1^ Å^−2^) harmonic restraints on solute heavy atoms. Unrestrained MD simulations followed for 5 ns at a temperature of 298 K and employed a 1 fs time step, giving a total of 0.5 μs of unrestrained simulation per system, accumulating 2 μs MD simulations in this study.

MD analyses, including pairwise contacts, backbone RMSF, and H-bond analysis based on cut-offs for the Donor-H···Acceptor distance (<2.4 Å) and angle (>120 deg), were performed with the MDtraj open library (45). All structural images were generated with PyMOL (available from https://www.pymol.org).

## Data availability

All data supporting the findings of this study are available within the paper and supporting information. Unprocessed NMR data will be made available upon request. Correspondence: matthew.eddy@ufl.edu.

## Supporting information

This article contains supporting information (4, 5, 17, 21, 46, 47, 48).

## Conflict of interest

The authors declare that they have no conflicts of interest with the contents of this article.
