# Supplementary material for: Interaction networks within disease-associated GαS variants characterized by an integrative biophysical approach
Source: J Biol Chem. 2024 Jun 24;300(8):107497. doi: 10.1016/j.jbc.2024.107497 (PMC11325797; doi:10.1016/j.jbc.2024.107497)
Supplement: Supplemental Figures S1–S10 and Tables S1 and S2 [file mmc1.docx]

**Supporting information**

**Interaction Networks within Disease-Associated Gα_S_ Variants Characterized by an Integrative Biophysical Approach**

Kara Anazia^1,‡^, Lucien Koenekoop^2,‡^, Guillaume Ferré ^1,3^, Enzo Petracco^1,4^, Hugo Gutiérrez-de-Teran^2 *^, Matthew T. Eddy^1,5 *^

^1^Department of Chemistry; University of Florida; Gainesville, FL, 32611; USA

^2^Department of Cell and Molecular Biology, Computational Biology and Bioinformatics, Uppsala University; Uppsala, 75105; Sweden

^3^Present address: Institut de Pharmacologie et de Biologie Structurale (IPBS), Université de Toulouse, CNRS, Université Toulouse III - Paul Sabatier (UT3), Toulouse, France

^4^URD Agro-Biotechnologies Industrielles (ABI), CEBB, AgroParisTech, Pomacle, France

^‡^These authors have contributed equally to this work

^5^Lead contact

*Correspondence: hugo.gutierrez@icm.uu.se, matthew.eddy@ufl.edu

**Supplementary results**

| **Table of contents** | **Page #** |
| --- | --- |
| Figure S1. Purification of Gα_S_ protein and functionality. | S-3 |
| Table S1. Summary of biochemical properties of Gα_S_ disease-causing variants | S-5 |
| Table S2. Primers designed for Gα_S_ variants | S-6 |
| Figure S2. Thermal melting profile of Gα_S_ and diseased variants in GDP and GTPγS determined by circular dichroism. | S-7 |
| Figure S3. Thermal melting profiles of Gα_S_ and Gα_S_ variants in the presence of GDP, GTPγS, or with no nucleotide added (apo). | S-8 |
| Figure S4. Backbone root mean square fluctuations (RMSF) values within the switch regions of Gα_S_ and Gα_S_ variants in complexes with GDP or GTP | S-9 |
| Figure S5. Non-bonded interaction energies for Gα_S_ and Gα_S_ variants. | S-10 |
| Figure S6. ^1^H signal assignment in reference spectra of GDP and GppNHp | S-11 |
| Figure S7. Residues closest to bound GTP in the Gα_S_ nucleotide binding pocket | S-12 |
| Figure S8. Optimization of STD-NMR saturation transfer time | S-13 |
| Figure S9. One-dimensional ^1^H STD-NMR spectra of Gα_S_ and Gα_S_ variants in complexes with GDP and GppNHp | S-14 |
| Figure S10. Percent of contact of residues of Gα_S_ and Gα_S_ variants with GDP and GTP protons in STD-NMR over MD simulations | S-15 |
| References | S-16 |


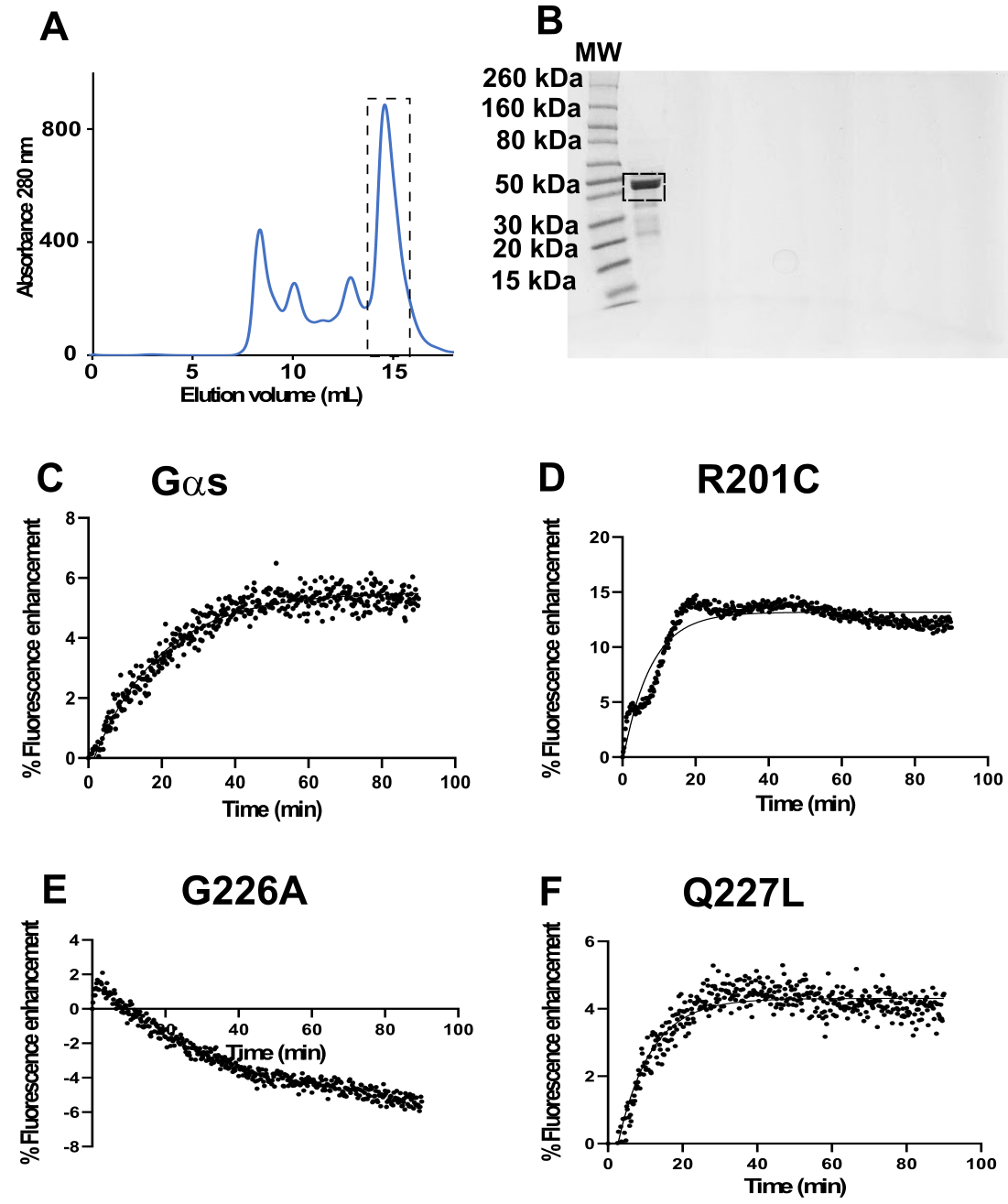


**Figure S1. Purification and functional assessment of Gα_S_** **and Gα_S_ variants.** *A*, size exclusion chromatogram of Gα_S_. The dashed box around the peak at 15 minutes corresponds to Gα_S_. Fractions in the center of this peak were selected for subsequent purification steps and analysis. *B,* SDS page gel of purified Gα_S_. The left lane shows molecular weight standards. The dashed box corresponds to the band for Gα_S_, consistent with its expected molecular weight of ~44kDa. *C-F,* tryptophan fluorescence assay of (C) Gα_S_ showing an increase in tryptophan fluorescence upon GTP binding and activation, and the Gα_S_ variants *(D)* R201C, (*E)* G226A, and *(F)* Q227L.

**Table S1 Summary of biochemical properties of Gα_S_** **disease-associated variants**

| **Gα_S_ disease variant** | **Disease presentation** | **Location in Gα_S_ structure** | **Activation of adenylyl cyclase** | **GDP dissociation**  **rate** | **GTP binding**  **rate** | **GTPase activity**  **rate** | **References** |
| --- | --- | --- | --- | --- | --- | --- | --- |
| R201C | Pituitary tumors and lung carcinomas | Switch I | Increased | Decreased | Decreased | Decreased | [1, 2] |
| Q227L | Pituitary tumors | Switch II | Increased | Decreased | Decreased | Decreased | [1, 3] |
| A366S | Testotoxicosis and Pseudohypoparathyroidism | loop connecting β6 and α5 | Increased | Increased | - | Increased | [4, 5] |
| R228C | Pseudohypoparathyroidism | Switch II | Decreased | Increased | Similar to Gα_S_ | Similar to Gα_S_ | [1, 2] |
| R258A | Albright’s Hereditary Osteodystrophy | Switch III | Decreased | Increased | Increased | Increased | [2, 6] |
| R265H | Albright’s Hereditary Osteodystrophy | Switch III | Decreased | Increased | Increased | Increased | [2] |
| G226A | Lymphoma | Switch II | Decreased | Similar to Gα_S_ | Similar to Gα_S_ | Decreased | [7] |

**Table S1. Summary of biochemical properties of Gα_S_ disease-associated variants.** Each variant is characterized in terms of which disease state it is represented in, the location in the Gα_S_ structure, the effect of the mutation on the activation of adenylyl cyclase, the rate of GDP dissociation, the rate of GTP binding and the rate of GTPase activity. The dash indicates that this value was not determined within the cited study.

**Table 2. Primers used to generate** **Gα_S_** **variants via site-directed mutagenesis.** Sequences of forward and reverse primers used to generate Gα_S_ variants from the original construct described in the Methods section.

| Mutation | Directionality | Primer |
| --- | --- | --- |
| R201C | Forward | GACTATGTGCCGAGCGATCAGGACCTGCTTCGCTGCTGCGTCCTGACTTCTGGAATCTTTGAG |
|  | Reverse | CTCAAAGATTCCAGAAGTCAGGACGCAGCAGCGAAGCAGGTCCTGATCGCTCGGCACATAGTC |
| G226A | Forward | CAACTTCCACATGTTTGACGTGGGTGCCCAGCGCGATGAACGCCGCAAGTGG |
|  | Reverse | CCACTTGCGGCGTTCATCGCGCTGGGCACCCACGTCAAACATGTGGAAGTTG |
| Q227L | Forward | TTCCACATGTTTGACGTGGGTGGCCTGCGCGATGAACGCCGCAAGTGGAT |
|  | Reverse | GATCCACTTGCGGCGTTCATCGCGCAGGCCACCCACGTCAAACATGTGGAA |
| R228C | Forward | CACATGTTTGACGTGGGTGGCCAGTGCGATGAACGCCGCAAGTGGATCCAG |
|  | Reverse | CTGGATCCACTTGCGGCGTTCATCGCACTGGCCACCCACGTCAAACATGTG |
| R258A | Forward | AGCAGCAGCTACAACATGGTCATCGCCGAGGACAACCAGACCAACCGCCTG |
|  | Reverse | CAGGCGGTTGGTCTGGTTGTCCTCGGCGATGACCATGTTGTAGCTGCTGCT |
| R265H | Forward | CATCCGGGAGGACAACCAGACCAACCATCTGCAGGAGGCTCTGAACCTCTTC |
|  | Reverse | GAAGAGGTTCAGAGCCTCCTGCAGATGGTTGGTCTGGTTGTCCTCCCGGATG |
| A366S | Forward | CTACTGCTACCCTCATTTCACCTGCTCTGTGGACACTGAGAACATCCGCCGTGTG |
|  | Reverse | CACACGGCGGATGTTCTCAGTGTCCACAGAGCAGGTGAAATGAGGGTAGCAGTAG |


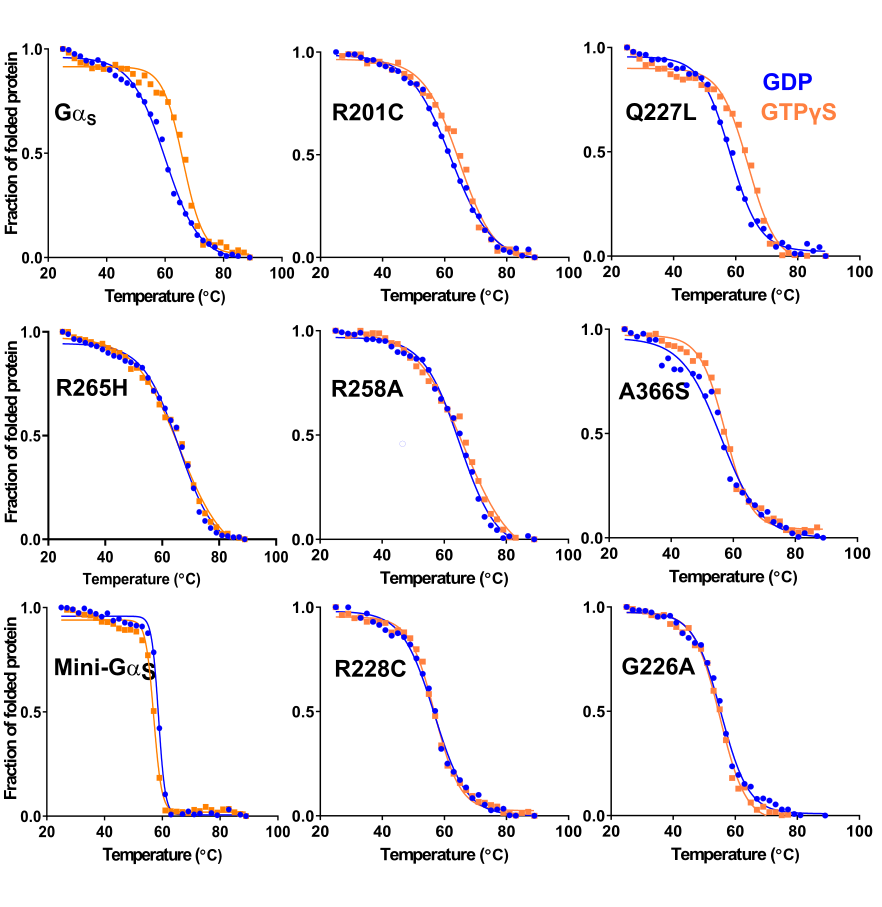
**Figure S2. Representative thermal melting curves of Gα_S_ and Gα_S_ variants in complex with either GDP or GTPγS, as determined by circular dichroism.** The thermal unfolding of Gα_S_ and Gα_S_ variants bound to GDP or GTPγS was monitored by variable temperature single wavelength CD, as shown in these representative plots. Same color scheme as shown in Figure 1*A*. The thermal unfolding temperatures (T_m_) were obtained by fitting data from triplicate experiments..

**Figure S3. Thermal melting profiles of Gα_S_ and Gα_S_ variants in the presence of GDP, GTPγS, or with no nucleotide added (apo).** *A,* Representative thermal unfolding curves of Gα_S_ and variants Gα_S_[R201C], Gα_S_[R258A] and Gα_S_[R265H] when bound to GDP, GTPγS or with no nucleotide added (apo), as monitored by variable temperature single wavelength CD. Same color scheme used as in Figure 1. *B,* histograms of the melting temperature (T_m_) values determined by fitting data from three independent experiments. Error bars represent the standard deviation of triplicate measurements.


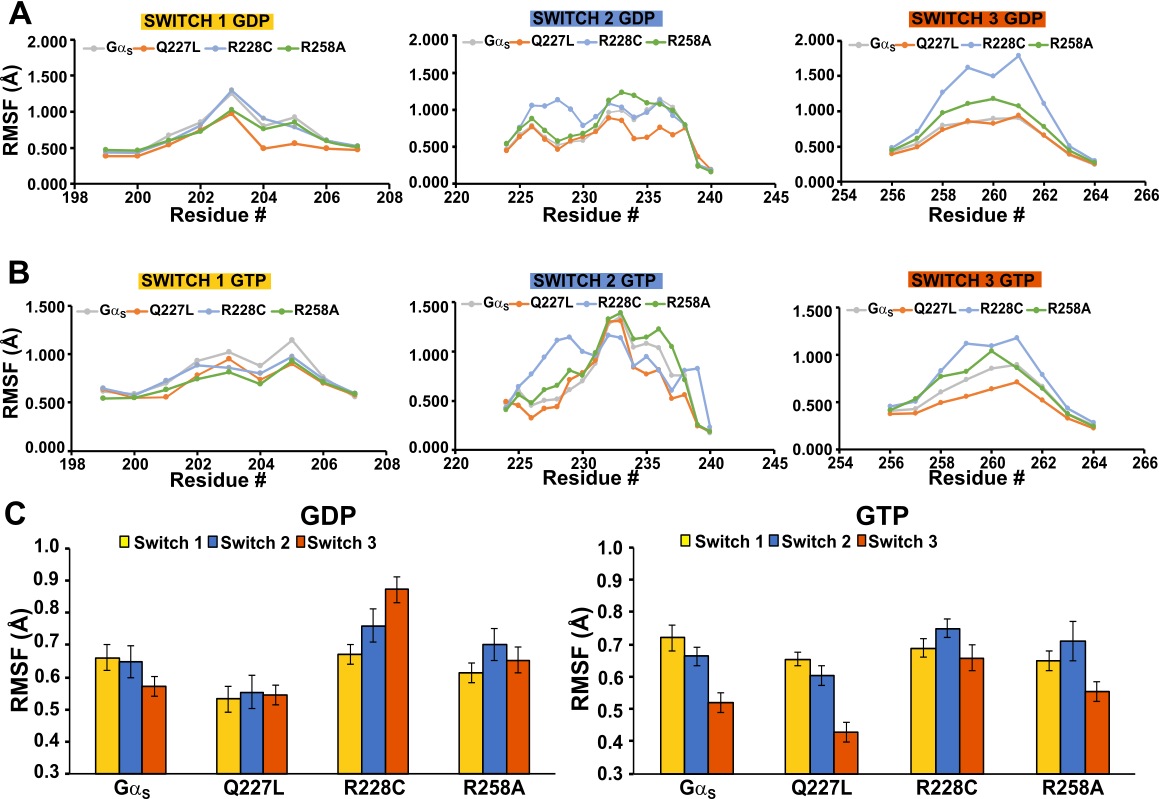


**Figure S4. Backbone root mean square fluctuations (RMSF) values within the switch regions of Gα_S_ and Gα_S_ variants in complexes with GDP or GTP**. *A* and *B,* line plots of backbone RMSF values of residues in switch I, II and III regions for Gα_S_ (gray), Gα_S_[Q227L] (orange), Gα_S_[R228C] (light blue) and Gα_S_[R258A] (green). *C,* histograms of the average RMSF values for Gα_S_ and the Gα_S_ variants bound to GDP (left panel) and GTP (right panel) in the regions of switch I (yellow bars), switch II (blue bars) and switch III (red bars). Error bars in the histograms represent the standard error of the mean.

**Figure S5. Non-bonded interaction energies for Gα_S_ and Gα_S_ variants.** Non-bonded interaction energies were averaged along the MD trajectories and normalized with respect to GTP-bound Gα_S_ (labeled “WT”) for both GDP-bound and GTP-bound variants.

**Figure S6. ^1^H signal assignment in reference spectra of GDP and GppNHp.** 1-dimensional ^1^H-NMR reference spectra of GDP (blue) and GppNHp (orange) nucleotides. ^1^H signals labeled ‘a’ through ‘d’ were utilized in STD-NMR experiments and are shown on the chemical structures of GDP and GppNHp. Assignments were transferred from BMRB entry bmse000270. ^1^H signals labeled 1 and 2 are from HEPES buffer, and ^1^H signals labeled 3-5 are from the sodium trimethylsilylpropanesulfonate (DSS) NMR standard.


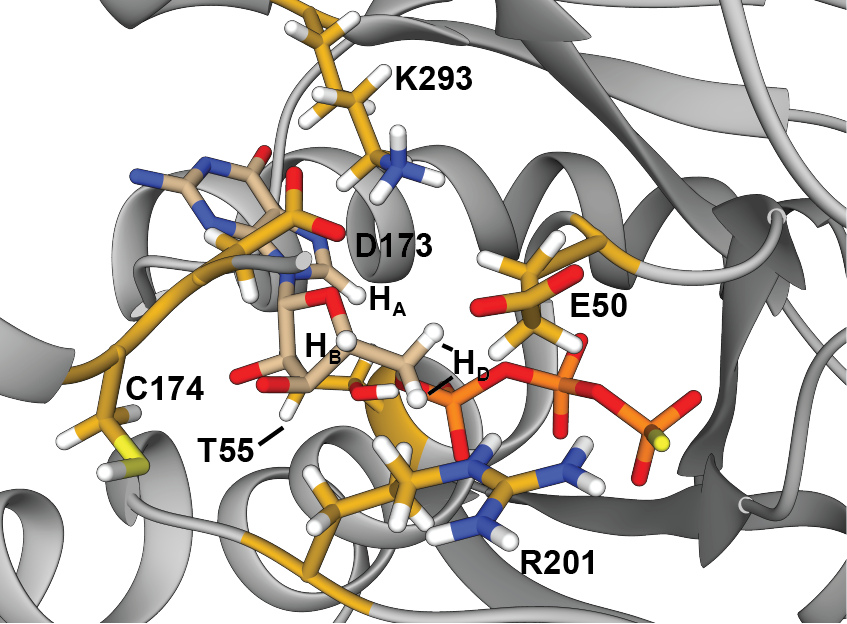


**Figure S7. Residues closest to bound GTP in the Gα_S_** **nucleotide binding pocket**. An expanded view is shown of the Gα_S_ nucleotide binding pocket with GTP bound (shown in tan stick representation; PDB:1AZT) with protons seen in STD-NMR interactions annotated ‘H_A_’ through ‘H_D_’. Residues within 6Å of these protons are annotated.


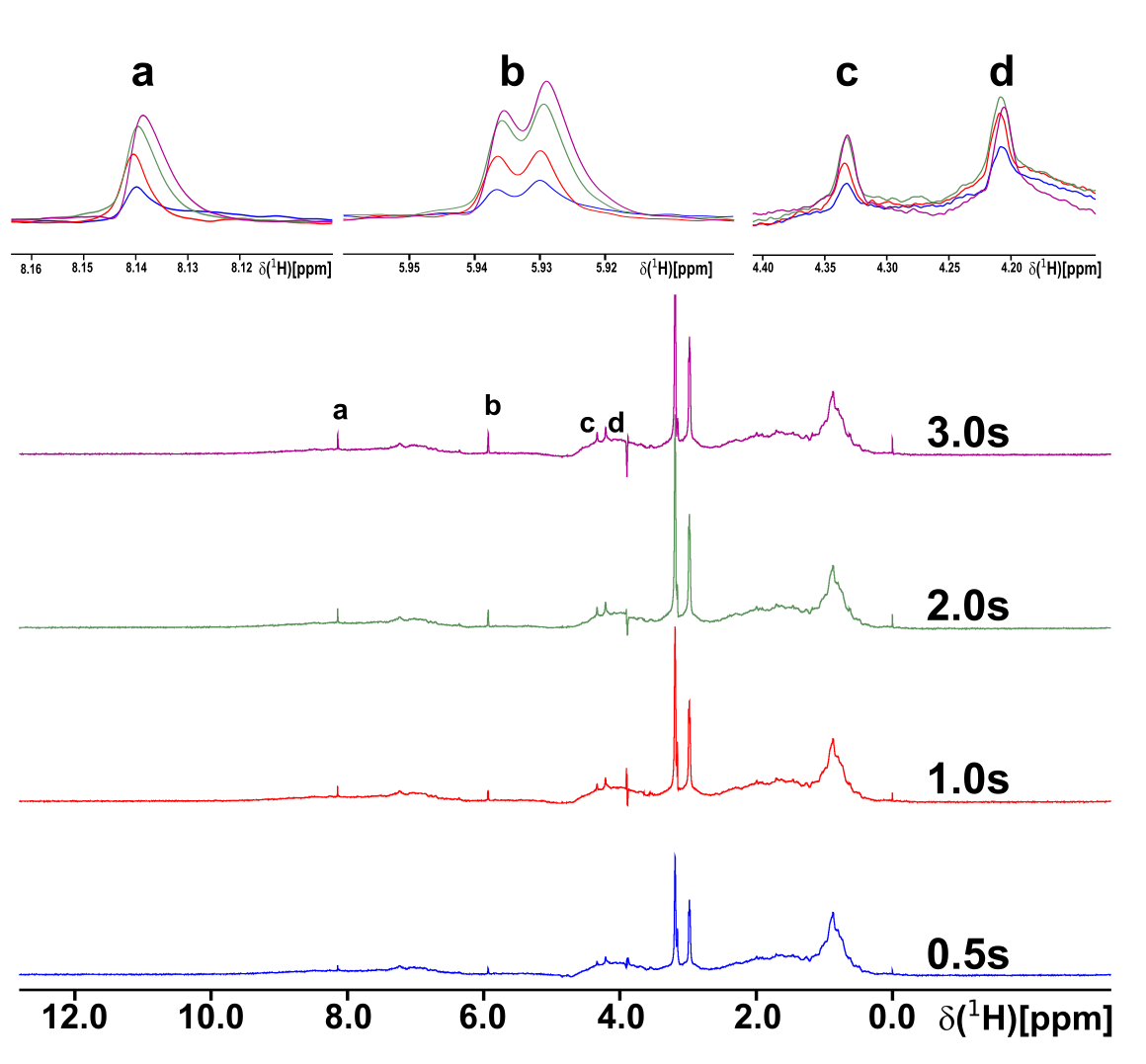


**Figure S8. Optimization of STD-NMR saturation transfer time.** ^1^H STD-NMR spectra are shown with 40 µM Gα_S_ and 2 mM GDP recorded with four different saturation transfer times between 0.5 s and 3.0 s, as indicated. ^1^H signals labeled ‘a’ through ‘d’ were used to calculate STD-NMR amplification factors. Expanded views of each signal are shown in the panels at the top.


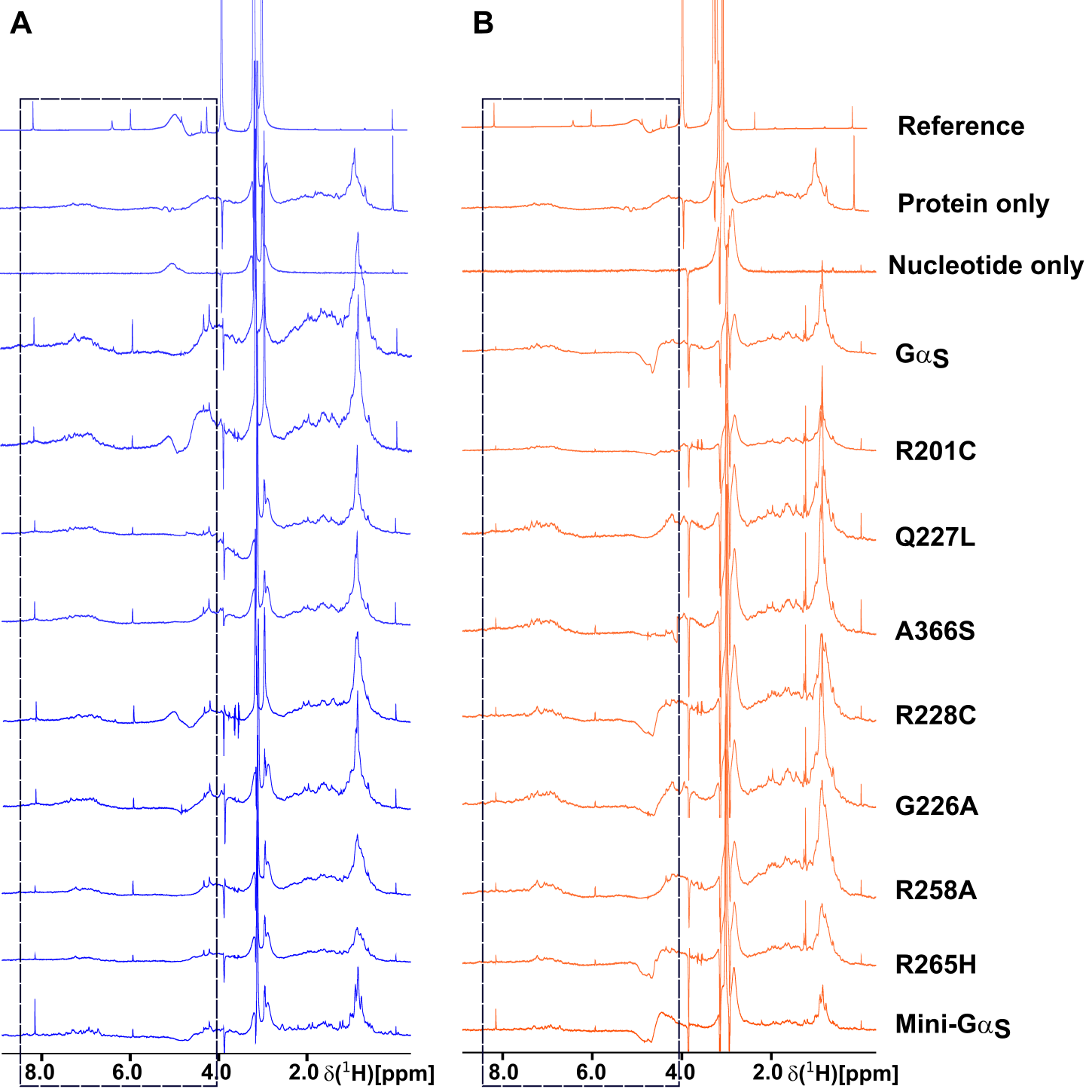


**Figure S9. One-dimensional ^1^H STD-NMR spectra of Gα_S_** **and Gα_S_ variants in complexes with GDP and GppNHp**. STD-NMR spectra of Gα_S_ and variants in complex with GDP shown in panel *A* (blue) and GppNHp shown in panel *B* (orange). “Reference” is a 1D ^1^H NMR spectrum of GDP, “protein only” is a STD-NMR control experiment with a sample containing Gα_S_ and buffer but no nucleotide, and “nucleotide only” is a STD-NMR control experiment with a sample containing nucleotide and buffer but no protein. Boxed regions indicate areas of interest where an STD-NMR effect is seen.


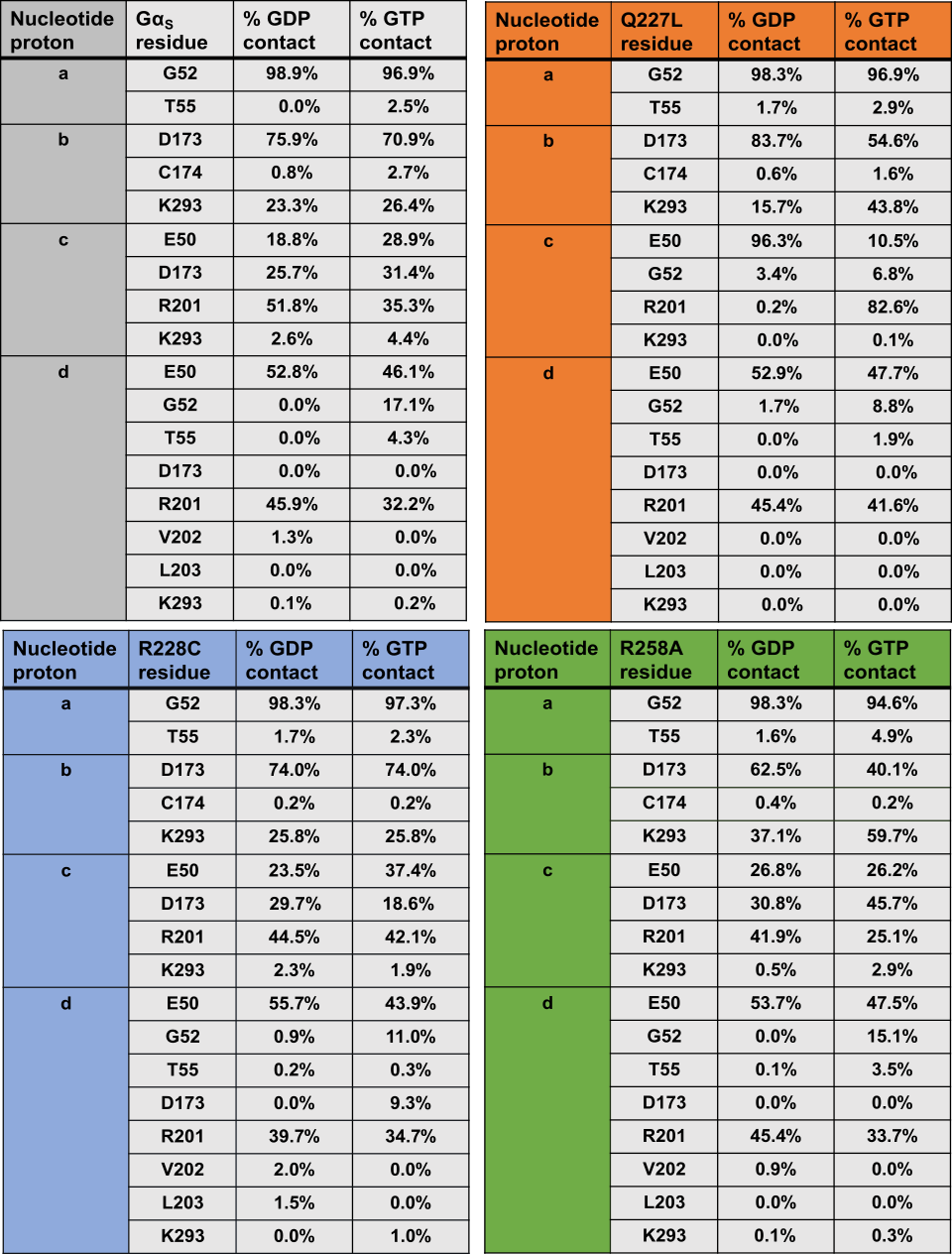


**Figure S10. Percent of contact of residues of Gα_S_ and Gα_S_ variants with GDP and GTP protons in MD simulations.** The percent contact of residues of the Gα_S_ protein and Gα_S_[Q227L], Gα_S_[R228C] and Gα_S_[R258A] in contact with the protons “a” to d” (d is the average of chemically equivalent protons) in GDP and GTP over the course of MD simulations.

**References**

1. O'Hayre, M., et al., *The emerging mutational landscape of G proteins and G-protein-coupled receptors in cancer.* Nature Reviews Cancer, 2013. **13**(6): p. 412-424.

2. Hu, Q. and K.M. Shokat, *Disease-Causing Mutations in the G Protein Gαs Subvert the Roles of GDP and GTP.* Cell, 2018. **173**(5): p. 1254-1264.

3. Graziano, M.P. and A.G. Gilman, *Synthesis in Escherichia coli of GTPase-deficient mutants of G_sα_.* Journal of Biological Chemistry, 1989. **264**(26): p. 15475-15482.

4. Taroh liri, P.H., Jon M. Nakamoto, Cornelis Van Dop and Henry R. Bourne, *Rapid GDP release from G_sα_ in patients with gain and loss of endocrine function.* Nature, 1994. **371**: p. 164-167.

5. Sun, D., et al., *Probing Gα_i1_ protein activation at single-amino acid resolution.* Nat Struct Mol Biol, 2015. **22**(9): p. 686-694.

6. Warner, D.R. and L.S. Weinstein, *A mutation in the heterotrimeric stimulatory guanine nucleotide binding protein α-subunit with impaired receptor-mediated activation because of elevated GTPase activity.* Proceedings of the National Academy of Sciences of the United States of America, 1999. **96**(8): p. 4268-4272.

7. Lee, E., R. Taussig, and A.G. Gilman, *The G226A mutant of G_sα_ highlights the requirement for dissociation of G protein subunits.* Journal of Biological Chemistry, 1992. **267**(2): p. 1212-1218.
